# Metagenomic and Metaproteomic Insights into Photoautotrophic and Heterotrophic Interactions in a *Synechococcus* Culture

**DOI:** 10.1128/mBio.03261-19

**Published:** 2020-02-18

**Authors:** Qiang Zheng, Yu Wang, Jiayao Lu, Wenxin Lin, Feng Chen, Nianzhi Jiao

**Affiliations:** a State Key Laboratory for Marine Environmental Science, Institute of Marine Microbes and Ecospheres, Xiamen University, Xiamen, People’s Republic of China; b College of Ocean and Earth Sciences, Xiamen University, Xiamen, People’s Republic of China; c Institute of Marine and Environmental Technology, University of Maryland Center for Environmental Science, Baltimore, Maryland, USA; Corporación CorpoGen - Research Institute

**Keywords:** *Synechococcus* culture, interactions, high-molecular-weight DOM, low-molecular-weight DOM, metagenome, metaproteome, exoproteome, transporters

## Abstract

Microbial photoautotroph-heterotroph interactions underlie marine food webs and shape ecosystem diversity and structure in upper ocean environments. Here, bacterial community composition, lifestyle preference, and genomic- and proteomic-level metabolic characteristics were investigated for an open ocean *Synechococcus* ecotype and its associated heterotrophs over 91 days of cocultivation. The associated heterotrophic bacterial assembly mostly constituted five classes, including *Flavobacteria*, *Bacteroidetes*, *Phycisphaerae*, *Gammaproteobacteria*, and *Alphaproteobacteria*. The seven most abundant taxa/genera comprised >90% of the total heterotrophic bacterial community, and five of these displayed distinct lifestyle preferences (free-living or attached) and responses to *Synechococcus* growth phases. Six high-quality genomes, including *Synechococcus* and the five dominant heterotrophic bacteria, were reconstructed. The only primary producer of the coculture system, *Synechococcus*, displayed metabolic processes primarily involved in inorganic nutrient uptake, photosynthesis, and organic matter biosynthesis and release. Two of the flavobacterial populations, *Muricauda* and *Winogradskyella*, and an SM1A02 population, displayed preferences for initial degradation of complex compounds and biopolymers, as evinced by high abundances of TonB-dependent transporters (TBDTs), glycoside hydrolase, and peptidase proteins. Polysaccharide utilization loci present in the flavobacterial genomes influence their lifestyle preferences and close associations with phytoplankton. In contrast, the alphaproteobacterium *Oricola* sp. population mainly utilized low-molecular-weight dissolved organic carbon (DOC) through ATP-binding cassette (ABC), tripartite ATP-independent periplasmic (TRAP), and tripartite tricarboxylate transporter (TTT) transport systems. The heterotrophic bacterial populations exhibited complementary mechanisms for degrading *Synechococcus-*derived organic matter and driving nutrient cycling. In addition to nutrient exchange, removal of reactive oxygen species and vitamin trafficking might also contribute to the maintenance of the *Synechococcus*-heterotroph coculture system and the interactions shaping the system.

## Abstract (precis)

The high complexity of *in situ* ecosystems renders it difficult to study marine microbial photoautotroph-heterotroph interactions. Two-member coculture systems of picocyanobacteria and single heterotrophic bacterial strains have been thoroughly investigated. However, *in situ* interactions comprise far more diverse heterotrophic bacterial associations with single photoautotrophic organisms. In the present study, combined metagenomic and metaproteomic data supplied the metabolic potentials and activities of uncultured dominant bacterial populations in the coculture system. The results of this study shed light on the nature of interactions between photoautotrophs and heterotrophs, improving our understanding of the complexity of *in situ* environments.

## INTRODUCTION

Interactions between photoautotrophic and heterotrophic bacteria shape the structure and diversity of upper ocean ecosystems and are foundational to marine food webs (1–3). Phytoplankton are significant contributors to primary production in aquatic environments, and marine picocyanobacteria primarily comprising *Prochlorococcus* and *Synechococcus* species account for almost half of the total ocean primary production (4, 5). Marine bacteria take up labile dissolved organic carbon (DOC) that is released by phytoplankton into surrounding waters, and they transform a fraction of this DOC into recalcitrant forms via a series of metabolic processes collectively referred to as the microbial carbon pump (6). During DOC mineralization by heterotrophic bacteria, inorganic nutrients (e.g., NH_4_^+^, NO_3_^−^, PO_4_^3−^, and Fe^2+^) that promote phytoplankton growth are regenerated. Thus, marine bacteria are important contributors to the global biogeochemical cycling of carbon, nitrogen, and phosphate (7, 8).

Interactions between photoautotrophs and heterotrophs in both natural environments and laboratory cultures have been extensively explored in recent decades (2, 8–10). Among these interactions, synergistic and antagonistic relationships have been commonly observed between photoautotrophs and heterotrophs. However, it is generally thought that photoautotrophs and heterotrophs develop mutualistic relationships through nutrient cycling. Specifically, photoautotrophs provide labile organic matter for heterotrophic growth, and in return, they benefit from the supply of essential micronutrients from heterotrophic bacteria, including vitamins and bioavailable trace metals (9, 11, 12), in addition to the removal of reactive oxygen species (ROS) (13, 14). In addition to mutualistic interactions, competition and antagonistic behaviors have also been observed between photoautotrophs and heterotrophs. For example, such interactions include competition for essential inorganic nutrients like phosphate and nitrate, in addition to the production of algicidal compounds by heterotrophic bacteria (15, 16).

Isolated marine unicellular cyanobacterial cultures, including those of *Synechococcus* and *Prochlorococcus*, typically contain coexisting heterotrophic bacterial partners (17, 18). Such associations between *Synechococcus* and heterotrophic bacteria have also been observed in natural environments (19). Although axenic cultures of *Cyanobacteria* can be achieved, *Synechococcus* and *Prochlorococcus* cultures with coexisting bacterial populations are more stable and have more robust and longer lifespans than axenic cultures (8, 13, 20). The interactions between picocyanobacteria and single heterotrophic bacterial strains (e.g., *Synechococcus*-Ruegeria pomeroyi DSS-3, *Synechococcus*-*Shewanella*, *Synechococcus*-*Vibrio*, and *Prochlorococcus*-*Alteromonas*) have been thoroughly investigated, yielding evidence for synergistic relationships among the two partners in coculture systems (8, 20–24). However, *in situ* interaction networks between photoautotrophs and heterotrophs are not only two-member systems but instead comprise diverse heterotrophic bacterial assemblies associated with a single photoautotrophic strain. These myriad relationships are not only synergistic in nature but also competitive and antagonistic.

The lifestyles and genomic characteristics of dominant heterotrophic bacteria associated with coastal *Synechococcus* sp. strain XM-24 cultures have been investigated in detail, but the associated heterotrophs also show geographic distribution patterns that are related to the environments of *Synechococcus* strain isolation (25, 26). Thus, several important aspects of these relationships remain unclear including (i) how open ocean *Synechococcus* culture ecotypes interact with their associated heterotrophic bacteria, (ii) how different heterotrophic bacterial populations interact with single cyanobacterial strains in coculture systems, and (iii) the nature of the roles of major bacterial populations in the *Synechococcus* culture systems. To address these gaps in knowledge, this study investigated (i) the assembly of culture-associated heterotrophic bacterial populations and their lifestyles across the growth period of an open ocean *Synechococcus* ecotype, (ii) the metabolic characteristics of the dominant bacteria in the coculture systems via metagenomic and metaproteomic analyses, and (iii) the potential associations of different bacterial populations in the context of the functioning and maintenance of coculture systems.

## RESULTS AND DISCUSSION

### Abundance variations and morphology of *Synechococcus* and associated heterotrophic bacteria.

**FIG 1:**
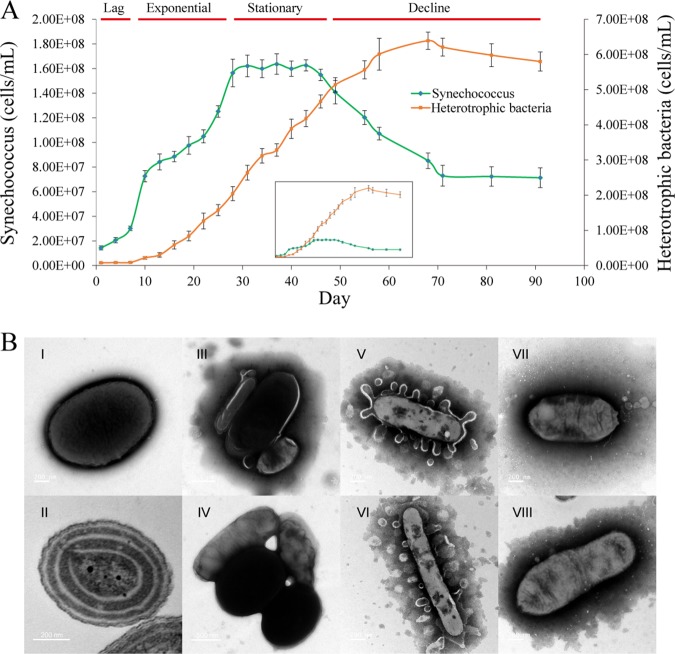
Abundance dynamics (A) and morphology (B) of *Synechococcus* and associated heterotrophic bacteria in the *Synechococcus* sp. strain YX04-3 coculture. The green curve represents *Synechococcus* cell abundances (left vertical axis) over time, while the orange curve shows heterotrophic bacterial cell abundances (right vertical axis) over time. The inset figure shows the dynamics among *Synechococcus* and heterotrophic bacterial abundances on the same vertical axis scale. Time (in days) is shown on the *x* axis. Error bars represent the ranges of values from triplicate measurements. The morphology of *Synechococcus* (I and II), attached cells or aggregates (III and IV), and associated heterotrophic bacteria (V to VIII) in the coculture system are depicted. Image scales are shown in the lower-left corner of the micrographs.

*Synechococcus* strain YX04-3 was isolated from the South China Sea using PRO2 liquid medium and purified after ∼5 to 7 rounds of dilution until a single *Synechococcus* internal transcribed spacer (ITS) sequence was obtained (25). This strain belongs to the clade III, subcluster 5.1 group of *Synechococcus* (see Fig. S1 in the supplemental material), and represents an open ocean ecotype. Its associated heterotrophs were a self-selected natural assemblage. During the 91-day incubation, no extra inorganic nutrients were added, and nutrient recycling maintained the coculture for a long time. The *Synechococcus* strain was inoculated at an initial density of 1.43 × 10^7^ cells ml^−1^ and plateaued at a population size of ∼1.60 × 10^8^ cells ml^−1^ during the experiment. Heterotrophic bacterial abundances ranged from the inoculated abundance of 7.78 × 10^6^ to 6.39 × 10^8^ cells ml^−1^ over the experiment. The *Synechococcus* growth curve exhibited four distinct phases comprising the lag, exponential, stationary, and decline phases (Fig. 1A). Phosphate was an important factor controlling *Synechococcus* growth in the culture (Fig. S2).

10.1128/mBio.03261-19.1 FIG S1 Neighbor-joining phylogenetic tree based on 16S-23S rRNA ITS sequences of representatives from cultured lineages of *Synechococcus*. Bootstrap percentages from neighbor-joining (1,000 replications) are shown. The scale bar represents a distance of 0.05 substitutions per site. The *Synechococcus* strains shown in red were isolated and collected by our lab. Download FIG S1, PDF file, 0.3 MB. Copyright © 2020 Zheng et al. 2020 Zheng et al. This content is distributed under the terms of the Creative Commons Attribution 4.0 International license.

10.1128/mBio.03261-19.2 FIG S2 Variation in phosphate (A) and organic carbon (B) concentrations in the coculture system over the incubation period. DOC, dissolved organic carbon; POC, particulate organic carbon. Error bars represent the ranges of values from triplicate measurements. Download FIG S2, PDF file, 0.3 MB. Copyright © 2020 Zheng et al. 2020 Zheng et al. This content is distributed under the terms of the Creative Commons Attribution 4.0 International license.

Transmission electron microscopy (TEM) was also used to investigate the morphological variation of *Synechococcus* and the heterotrophic bacterial cells, in addition to possible associations of the taxa in the coculture system. TEM revealed that *Synechococcus* cells exhibited short rod shapes (1.10 ± 0.10 μm by 0.85 ± 0.10 μm) with relatively strong electron densities (Fig. 1B, panels I and II). In addition, aggregated or attached cells with different morphologies were frequently observed in the coculture system. It seems that some aggregates comprised *Synechococcus*-like cells in the centers and attached heterotrophic bacteria at the periphery (Fig. 1B, panels III and IV). Bacteria exhibiting long rod shapes (0.20 ± 0.05 μm by 2.0 ± 0.50 μm) released considerable numbers of vesicles (Fig. 1B, panels V and VI). In addition, other bacteria with short rod shapes (0.40 ± 0.10 μm by 1.20 ± 0.20 μm) exhibited mouth-like pit structures at cell ends that could be channels related to the uptake of macromolecular organic matter (Fig. 1B, panels VII and VIII) (27, 28).

### Microbial community assembly in the *Synechococcus* sp. strain YX04-3 coculture.

**FIG 2:**
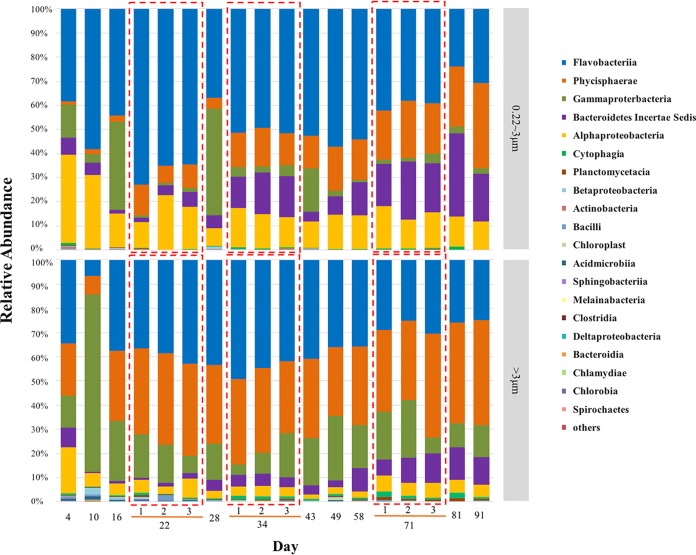
Heterotrophic bacterial community structure from the 0.22-to-3-μm (top panel) and >3-μm (bottom panel) size fractions over the duration of the cultivation experiment. The relative abundances of different bacterial classes are based on the total heterotrophic bacterial 16S rRNA sequence abundances after removing *Synechococcus* sequences for each time point from the size-fractionated samples. Triplicate samples from the 0.22-to-3- μm and >3-μm size fractions were sequenced for the 22nd, 34th, and 71st day of cultivation (shown in red boxes).

**FIG 3:**
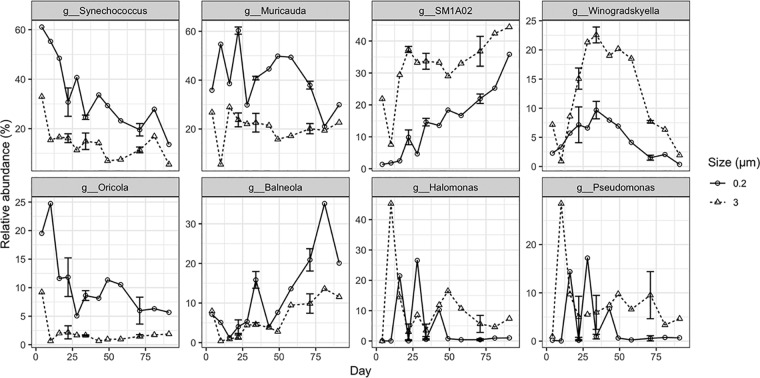
Relative abundances of *Synechococcus* (g_Synechococcus) in the total bacterial 16S rRNA sequence data sets and the seven dominant heterotrophic bacterial taxa among the total heterotrophic bacterial sequences from the 0.22-to-3-μm and >3-μm size fractions over the cultivation experiment. g_Synechococcus, genus *Synechococcus*, g__Muricauda, genus *Muricauda*, g__Winogradskyella, genus *Winogradskyella*, g__SM1A02, group SM1A02 belonging to family *Phycisphaeraceae*, g__Oricola, genus *Oricola*, g__Balneola, genus *Balneola*, g__Halomonas, genus *Halomonas*, g__Pseudomonas, genus *Pseudomonas*. Detailed descriptions of the lifestyle strategies of the dominant bacterial populations of the coculture are provided in Text S1 in the supplemental material.

To evaluate the prevalence of different lifestyles (i.e., free-living versus attached/aggregated) among cells in the *Synechococcus* sp. strain YX04-3 coculture, 16S rRNA amplicons from 36 samples were amplified and sequenced over the duration of the culture incubation from two different size fractionation groups. A total of 1,271,032 16S rRNA gene sequences (617,028 sequences from the 0.22-to-3-μm size fractions and 654,004 sequences from the >3-μm size fractions) were generated after quality control. The average relative abundances of *Synechococcus* in the total 16S rRNA sequences for each size fraction represented 30.2% and 12.9% of the total bacteria in the 0.22-to-3-μm and >3-μm size fractions, respectively, over the entire culture growth period (Fig. 2 and 3). However, it should be noted that the relative abundances of *Synechococcus* are heavily underestimated due to multiple 16S rRNA gene copies in the genomes of the dominant heterotrophic bacteria in the coculture system (see Table S1A in the supplemental material). Consequently, we primarily focus on the distributional patterns among the different size fractions. After removal of *Synechococcus* sequences, a total of 430,701 and 569,501 heterotrophic bacterial sequences were obtained from the 0.22- and 3-μm size fractions, respectively. The heterotrophic bacterial 16S rRNA gene sequences primarily constituted five classes: *Flavobacteriia* (31.4% of the total 16S rRNA gene sequences), *Phycisphaerae* (20.7%), *Gammaproteobacteria* (11.1%), *Bacteroidetes* (6.8%), and *Alphaproteobacteria* (6.6%) (Fig. 2).

10.1128/mBio.03261-19.7 TABLE S1 (A) 16S rRNA gene copy numbers in the complete genomes of close relatives of the strains whose genomic bins were recovered in this study. (B) Proteins identified in the proteomes of each coculture population. Download Table S1, DOC file, 0.04 MB. Copyright © 2020 Zheng et al. 2020 Zheng et al. This content is distributed under the terms of the Creative Commons Attribution 4.0 International license.

10.1128/mBio.03261-19.10 TEXT S1 Genomic characteristics and lifestyle strategies of the dominant bacterial populations of the coculture, *Synechococcus* sp. YX04-3 coculture system exoproteome, supplemental Materials and Methods, and supplemental references. Download Text S1, DOC file, 0.08 MB. Copyright © 2020 Zheng et al. 2020 Zheng et al. This content is distributed under the terms of the Creative Commons Attribution 4.0 International license.

The seven most abundant heterotrophic genera/taxa, including *Muricauda*, SM1A02 group, *Winogradskyella*, *Oricola*, *Balneola*, *Halomonas*, and *Pseudomonas*, comprised more than 90% of the total heterotrophic bacterial 16S rRNA gene sequences within each size fraction. Comparison of taxonomic abundances across time points and size fractions revealed distinct patterns of lifestyle preference (free-living versus attached) and variation with *Synechococcus* growth phases (Fig. 3). The relative abundances of the *Muricauda* and *Oricola* genera from the *Flavobacteriia* and *Alphaproteobacteria* classes, respectively, were significantly higher in the 0.22-to-3-μm size fractions than in the >3-μm size fraction (*t* test, *P* < 0.01) (Fig. 3 and Table 1). In contrast, the relative abundances of the SM1A02 group and the *Winogradskyella* genus that belong to the *Phycisphaerae* and *Flavobacteriia* classes, respectively, were significantly higher in the >3-μm size fraction than in the 0.22-to-3-μm size fraction (*P* < 0.01) (Fig. 3 and Table 1). The *Balneola* genus exhibited increased relative abundances after the 50th day of cultivation (Fig. 3 and Table 1). In contrast, the gammaproteobacterial genera *Halomonas* and *Pseudomonas* did not exhibit lifestyle preferences or significant responses to *Synechococcus* growth phases (Fig. 3).

**TABLE 1:** Characteristics of reconstructed genome bins and their relative abundances

Bin ID^*a*^	Taxonomy (class)	No. of contigs	Assem. size^*b*^ (Mbp)	GC content (%)	Est. comp.^*c*^ (%)	Est. cont.^*d*^ (%)	% relative abundance based on 16S rRNA gene sequences^*e*^					
							0.22 μm (D22)	3 μm (D22)	0.22 μm (D71)	3 μm (D71)	0.22 μm (avg)	3 μm (avg)
*Synechococcus* sp. YX04-3 (Bin1)	*Cyanobacteria*	16	2.46	59.3	99.50	0.00	30.73	16.14	19.56	11.40	34.00	14.15
*Muricauda* sp. Bin2	*Flavobacteria*	5	3.1	42	99.00	1.54	41.83	19.94	30.51	17.82	26.91	17.47
*Winogradskyella* sp. Bin3	*Flavobacteria*	10	3.15	33.8	99.20	2.47	5.12	12.61	1.20	6.81	3.17	10.80
*Phycisphaera* sp. Bin4	*Phycisphaerae*	1	3.26	61.2	98.90	0.00	6.93	31.36	17.68	32.57	10.41	27.57
*Oricola* sp. Bin5	*Alphaproteo-* *bacteria*	7	3.63	64.8	99.20	0.39	8.03	1.82	4.77	1.31	6.49	1.67
*Balneola* sp. Bin6	*Flavobacteria*	11	3.65	40	100	0.00	2.70	1.19	16.87	8.75	8.46	5.09

a ID, identifier.

b Assem. size, assembly size.

c Est. comp., estimated completeness.

d Est. cont., estimated contamination.

e Percent relative abundance based on 16S rRNA gene sequences from 0.22-to-3-μm and >3-μm size fractions on day 22 (D22) and 71 (D71) of cultivation. The average information (avg) for 36 samples collected from two size fractions, 0.22-to-3-μm and >3-μm size fractions are shown in the two rightmost columns.

### Metagenomic analysis of coculture communities.

Two samples from the exponential (22nd day) and decline (71st day) phases of the *Synechococcus* sp. YX04-3 culture were subjected to shotgun metagenomics sequencing on the Illumina MiSeq platform. Assembly of the metagenomic reads yielded 155 and 3,395 contigs (>1 kbp), comprising total assembly sizes of 15.7 and 24.2 Mbp, respectively. Six high-quality genomes (estimated genome completeness of >95% and estimated contamination of <5%) from the *Synechococcus* coculture were reconstructed from the assembled contigs, and are referred to as genomes Bin1 to Bin6 (Table 1). Five heterotrophic bacterial genomes (*Muricauda* sp. Bin2, *Winogradskyella* sp. Bin3, *Phycisphaera* sp. Bin4, *Oricola* sp. Bin5, and Balneola sp. Bin6) represented three bacterial phyla and corresponded to the dominant bacterial genera/groups of the cultivation community, with the exception of two gammaproteobacterial genera that were not represented.

These five dominant heterotrophic bacterial populations were closely related to strains reported to have close associations with marine phytoplankton or that otherwise exhibited the capacity to degrade algal polysaccharides. *Muricauda* can be abundant within diverse phytoplankton blooms or cultures, such as those produced by diatoms and dinoflagellates (29, 30). Further, Muricauda ruestringensis has been reported to utilize diverse carbohydrates and amino acids for growth (31). The flavobacterial genus *Winogradskyella* has been shown to associate with Cochlodinium polykrikoides and is a dominant particle-attached bacterial taxa (32). Winogradskyella rapida SCB36T was isolated from a seawater mesocosm experiment after the addition of protein and exhibited the most pronounced positive growth response to protein enrichment (33). Moreover, planctomycetes have frequently been observed to associate with marine phytoplankton blooms (34–39). Planctomycetes can exhibit diverse lifestyle strategies, including motility, unicellular stages, and aggregate formation with variable numbers of cells that are stacked with a fibrillar glycoproteic material known as the holdfast (40). Oricola cellulosilytica CC-AMH-0T is the only known member of the genus and has been previously shown to degrade cellulose and become particularly active under nutrient-limited conditions (41). *Balneola* has also been frequently isolated from phytoplankton cultures and particularly from those of Emiliania huxley and *Cochlodinium polykrikoides* (42, 43).

Detailed descriptions of genomic characteristics and lifestyle strategies of the dominant bacterial populations of the coculture are provided in Text S1 in the supplemental material.

### Cellular metaproteomic analyses and inference of metabolic potential.

The metagenomic data could be used to predict the metabolic potentials of dominant bacterial populations, while the metaproteomic data could offer insights into the real metabolic activities occurring in the coculture system, improving our understanding of photoautotroph-heterotroph interactions. A total of 2,047 proteins were identified through cellular metaproteomic analyses (Tables S1B and S2). Proteins from the *Synechococcus* (Bin1) and *Oricola* (Bin5) populations comprised 43.2% and 43.8% of the total coculture proteomic profile, respectively. The considerable differences in protein abundances from *Synechococcus* (Bin1) and *Oricola* (Bin5) populations compared to the three *Flavobacteria* and *Phycisphaera* populations reflect differences in their trophic and lifestyle strategies during the exponential phase. A very low level of proteins was recovered from the Bin6 population, and thus, the other five bins were primarily used for further analysis. To constrain our investigation to the potential interactions within the coculture system, functional proteins that are involved in transmembrane transport and utilization of nutrients and in the storage of carbon and energy sources were specifically analyzed.

10.1128/mBio.03261-19.8 TABLE S2 Cellular protein information for the proteomes of the five population bins of this study. Download Table S2, XLS file, 0.6 MB. Copyright © 2020 Zheng et al. 2020 Zheng et al. This content is distributed under the terms of the Creative Commons Attribution 4.0 International license.

### Proteomic profile of *Synechococcus* sp. YX04-3 (Bin1).

A total of 884 proteins that were attributable to *Synechococcus* in the coculture system were identified. Among these proteins, 27.48% of *Synechococcus* proteome (73 proteins) was dedicated to CO_2_ fixation and photosynthesis. A considerable relative abundance of the *Synechococcus* proteome was assigned to cell division (2.27% of *Synechococcus* proteome, 24 proteins), tRNA aminoacylation (1.26%, 21 proteins), ribosomal proteins (4.61%, 33 proteins), and translation factors (1.19%, 11 proteins). These recoveries were likely due to sampling during the exponential phase and the high metabolic and growth activity of *Synechococcus* during this time.

**(i) Two-component regulatory systems and nutrient uptake.** Bacteria typically use two-component regulatory systems comprising a sensor kinase and a response regulator to sense and respond to their environments (44). Three sensor histidine kinases and six response regulators were present in the proteomic profile of *Synechococcus*. Two of these were predicted to be involved in the phosphate sensor-regulatory (PhoRB) system. A total of 8 sensor histidine kinases and 11 response regulators were detected in the *Synechococcus* sp. YX04-3 genome, which is intermediate between those in the genomes of the oligotrophic *Synechococcus* strain WH8102 and the coastal strain CC9311 (45, 46). A lower abundance of sensors than response regulators is commonly observed in the genomes of *Synechococcus* and *Prochlorococcus* strains, which may represent an efficient regulation strategy wherein one sensor transmits signals to multiresponse regulators (44, 45).

*Synechococcus* requires sufficient nitrogen and phosphorus nutrients for growth. Accordingly, nitrate/nitrite transporters and reductases were identified in the *Synechococcus* proteome. Further, two abundant proteins, glutamine synthetase (0.43%) and glutamate synthase (0.02%), that are components of the primary bacterial pathway of ammonium assimilation were also identified. In addition, proteins that conferred the capacity for cyanate hydrolysis, including an ABC transporter system and a cyanase that catalyzes the metabolism of cyanide to CO_2_ and NH_3_, were recovered. Interestingly, several proteins involved in urea transport (UrtABCDE) and assimilation (UreCG) were detected, and they represented 0.84% of the *Synechococcus* proteome. Despite the fact that nitrate was the major N source provided in the medium, the urea ABC transporter protein was the most abundant inorganic nitrogen transporter protein observed, which is consistent with previous results (11, 47). Accordingly, a full complement of genes encoding a urea transporter system (*urtABCDE*) and assimilation pathway (*ureABCDEFG*) were present in the *Synechococcus* genome. The urea cycle is an important pathway of N recycling in *Synechococcus* cells, wherein urea, and eventually cyanate, are generated from arginine degradation (46).

In addition to nitrogen, phosphorus is a critical limiting nutrient for microbial growth in marine environments. High-affinity phosphate transporter (PstBS) and regulatory system (PhoBR) proteins were detected, and they accounted for 0.51% of the *Synechococcus* proteome. In addition, two phosphate starvation-inducible proteins (PhoH) were also observed on the 22nd day of cultivation, despite the fact that there was enough phosphate to support *Synechococcus* growth (Fig. S1). Iron (Fe) is another important limiting nutrient in the open ocean, and its transporter, one iron-binding ABC transport protein, was also identified in the proteomic profile of *Synechococcus*.

Amino acid and oligopeptide ABC transporters were also detected in the *Synechococcus* proteomic profile, indicating that the *Synechococcus* strain could utilize some low-molecular-weight (LMW) organic matter compounds (48). In addition, the genome of strain YX04-3 encoded proteins that could degrade chemically stable C-P bond-containing phosphonates (PhnCDE).

**(ii) Photosynthesis and glycogen metabolism.** Approximately 10% of the recovered *Synechococcus* proteins comprising 27.48% of the *Synechococcus* proteome were associated with CO_2_ fixation (5.75% of *Synechococcus* proteome, 10 proteins) and photosynthesis, including photosystem I (1.67%, 9 proteins) and II (3.22%, 13 proteins) assembly, chlorophyll (0.67%, 11 proteins) and carotenoid (0.36%, 7 proteins) biosynthesis, and phycobilisome (15.82%, 23 proteins) assembly (Fig. S3). A previous analysis of a close relative of strain YX04-3, *Synechococcus* sp. strain WH8102, under low light conditions indicated that it increased the expression of photosynthesis genes involved in light harvesting, phycobilisome assembly, and carbon fixation under these conditions (47). In addition to proteins involved in photosynthesis, seven proteins involved in glycogen biosynthesis and degradation (GlgABCDPX-MalZ) were detected and represented 0.87% of the *Synechococcus* proteome. *Synechococcus* can accumulate surplus photosynthetically fixed carbon to synthesize glycogen, which has been observed as granules in their cytoplasm (49).

10.1128/mBio.03261-19.3 FIG S3 Overview of the dominant metabolic processes of *Synechococcus* sp. YX04-3 including transporter systems and carbon, nitrogen, and phosphorus metabolisms in the proteome. The primary metabolic processes observed for strain YX04-3 were involved in inorganic nutrient (e.g., NO_3_^−^, PO_4_^3−^, and cyanate) uptake, photosynthesis, organic matter (e.g., glycogen) biosynthesis, and organic compound [e.g., polysaccharides, lipopolysaccharide, lipids and (oligo)peptide] release. VB12, vitamin B12; SOD, superoxide dismutase. The background arrows indicate the potential material and energy flows detected in the proteomic data. Download FIG S3, PDF file, 0.4 MB. Copyright © 2020 Zheng et al. 2020 Zheng et al. This content is distributed under the terms of the Creative Commons Attribution 4.0 International license.

**(iii) Export and secretion systems.** Several proteins were identified in high abundance (0.48% of the *Synechococcus* proteome) within the *Synechococcus* proteome that are involved in the secretion of organic compounds like lipids, polysaccharides, and proteins (Fig. S3). For example, proteins responsible for lipid A biosynthesis (LpxABCD, KtdA, and KdsD) and export (MsbA) were present in its proteomic profile. In addition, polysaccharide/lipopolysaccharide biosynthesis and export proteins were also abundant (0.5%) in its proteomic profile. Further, protein-export-related membrane proteins (SecADF) were abundant in the *Synechococcus* proteomic data, and especially the ATP-hydrolyzing SecA protein (0.11%). The release of organic matter is critical for heterotrophic bacterial growth that is associated with *Synechococcus* growth. Several proteins in the ABC and major facilitator superfamily group transporters were identified that are involved in the export of metals (e.g., lead, cadmium, zinc, and mercury), multidrugs, and toxins. These efflux transporter proteins could be involved in antagonistic interactions with cocultured heterotrophic bacteria via the export of toxins (44, 50).

### Flavobacterial proteomic profiles (bins 2 and 3).

A total of 71 and 22 proteins were identified as belonging to the *Flavobacteria* Bin2 and Bin3 populations, respectively (Tables S1B and S2 ). These proteins were primarily involved in the transport and metabolism of complex high-molecular-weight (HMW) organic compounds in addition to supporting attached/aggregate lifestyles (Fig. S4).

10.1128/mBio.03261-19.4 FIG S4 Overview of the dominant metabolic processes associated with the two *Flavobacteria* population bins in their proteomes. The primary observed processes were involved in the degradation and transport of high-molecular-weight (dissolved) organic matter in Bin2 (left) and Bin3 (right). These activities were mediated by TonB-dependent transport systems and polysaccharide (or polymer) utilization loci (PUL) complexes. The polysaccharide utilization system of the figure was modified from Flint et al. (88). SOD, superoxide dismutase. The background arrows indicate the potential material and energy flows detected in the proteomic data. Download FIG S4, PDF file, 0.4 MB. Copyright © 2020 Zheng et al. 2020 Zheng et al. This content is distributed under the terms of the Creative Commons Attribution 4.0 International license.

**(i) Biopolymer transport and utilization.** TonB-dependent transporters (TBDTs) were abundant proteins identified in the proteomic profiles of Bin2 (9 proteins) and Bin3 (5 proteins), comprising 10.95% and 19.37% of their proteomes, respectively. In particular, three SusCD protein complexes (defined here as A, B, and C) were detected in the Bin2 profile that are core components of TBDT systems and are responsible for the binding and uptake of biopolymers. In addition, three other SusC-like proteins were identified in the Bin2 proteome. A single SusCD protein complex (defined as D) that is encoded by the α-1,4-glucan utilization gene cluster was present in the Bin3 proteome, in addition to three other SusC-like proteins. Two glycohydrolase (GH) proteins and four peptidase proteins that are involved in the degradation and utilization of complex organic compounds were identified in the Bin2 proteome, with three and two such proteins identified in the Bin3 proteome, respectively.

**(ii) Proteins involved in adhesion or aggregation.** Two important outer membrane porin proteins, OmpA and OmpH (51, 52), were detected in the Bin2 proteomic profile, while only OmpA was observed in the Bin3 proteomic profile. OmpA is thought to specifically interact with host receptor molecules (53) and exhibits important roles in bacterial adhesion and aggregation (52). Tetratricopeptide repeat proteins (TRP) that are involved in protein-protein interactions were also present in both flavobacterial proteomes, and could be involved in the adhesion or aggregation of *Flavobacteria* in the coculture system (54, 55). Although 15 gliding motility genes were present in both flavobacterial genomes, their encoded proteins were not identified in the proteomic data.

### *Phycisphaera* sp. Bin4 proteomic profile.

The proteomic profile corresponding to *Phycisphaera* sp. Bin4 comprised 173 proteins, with 15.6% assigned to unknown function (i.e., hypothetical proteins). High abundances of peptidases and type IV pilus proteins in the Bin4 profile indicated the physiological potential for biopolymer degradation and motility. Seventeen peptidases, including a serine protease, glutamic protease, metalloprotease, and asparaginase, were identified and represented 6.74% of the *Phycisphaera* sp. Bin4 proteome, suggesting an enhanced ability to hydrolyze peptide bonds of proteins. Some secreted proteases can degrade extracellular proteins into amino acids that are important in nitrogen acquisition from proteins. The *Phycisphaera* sp. Bin4 population exhibited a preference for the utilization of peptide polymers and could mediate the supply of LMW DOC such as amino acids to the *Synechococcus* and *Oricola* sp. Bin5 populations.

### *Oricola* sp. Bin5 proteomic profile.

A total of 896 proteins were identified that were produced by the *Oricola* sp. Bin5 population, of which 555 were involved in biological processes, 196 were associated with cellular components, and 618 were involved in molecular functions under the Gene Ontology (GO) category. That indicates *Oricola* sp. Bin5 was the most active heterotrophic bacteria in the coculture system, although its abundance was not the highest on day 22. A previous study also proved that alphaproteobacteria, especially members of *Roseobacter*, were better “helper” for *Synechococcus* growth than other heterotrophic bacteria (8). Remarkably, approximately 100 transporters that are involved in the uptake of LMW DOC and inorganic nutrient uptake/transport were present in the Bin5 proteomic profile (Fig. S5).

10.1128/mBio.03261-19.5 FIG S5 Overview of the dominant metabolic processes associated with the *Oricola* sp. Bin5 population in the proteome. The dominant processes identified were associated with transporter systems and carbon, nitrogen, and phosphorus metabolisms. *Oricola* sp. Bin5 primarily imported low-molecular-weight organic substrates, including amino acids, di-/oligopeptides, nucleosides, ribose, maltose, xylose, dihydroxyacetone, C4-dicarboxylates, pyruvate, and sialic acids, as well as inorganic nutrients like NO_3_^−^ and PO_4_^3−^ using ATP-binding cassette (ABC), tripartite ATP-independent periplasmic (TRAP), and tripartite tricarboxylate transporters (TTT) transport systems. VB12, vitamin B12; SOD, superoxide dismutase. The background arrows indicate the potential material and energy flows detected in the proteomic data. Download FIG S5, PDF file, 0.3 MB. Copyright © 2020 Zheng et al. 2020 Zheng et al. This content is distributed under the terms of the Creative Commons Attribution 4.0 International license.

**(i) Polyhydroxyalkanoate metabolism.** Polyhydroxyalkanoate (PHA) is an intracellular carbon and energy storage polyester, and its accumulation and degradation are typically related to microbial growth in response to various environmental conditions like nutrient levels (56, 57). The key enzymes for PHA biosynthesis (i.e., PHA polymerase, PhaC) and degradation (i.e., PHA depolymerase, PhaZ) were present in the proteomic profile of *Oricola* sp. Bin5. Thus, *Oricola* sp. Bin5 cells were likely accumulating intracellular PHA in the rich organic matter environment of the coculture system.

**(ii) Transport systems and nutrient uptake.** A considerable number of transport proteins, including ATP-binding cassette (ABC) transporters and tripartite ATP-independent periplasmic (TRAP) transporters, were identified in the proteomic profile of *Oricola* sp. Bin5. A total of 87 ABC transporter proteins were identified, and they comprised 11.54% of the *Oricola* sp. Bin5 proteome, in which ∼80% were involved in the transport of LMW organic matter compounds, including amino acids, di-/oligopeptides, and carbohydrates (including glycerol-3-phosphate) (Table S2). Among these proteins, ABC transporter proteins involved in carbohydrate transport and uptake were the most abundant (36 proteins, 5.47% of the *Oricola* sp. Bin5 proteome). In particular, transporters of LMW carbohydrates, including xylose, fructose, nucleoside, ribose, maltose, polyols, dihydroxyacetone, and glycerol-3-phosphate were prevalent. In addition, nine proteins (1.68%) were identified in the Bin5 proteome that are associated with general ABC transporters and those for polar and branched-chain amino acids, in addition to 15 ABC transporters (1.92%) involved in di-/oligopeptide transport. An additional nine ABC transporter proteins with inferred roles in transporting putrescine, pyrimidine, and thiamine were also observed in the proteome. Further, 10 TRAP transporter proteins (2.22%) involved in transporting C4-dicarboxylates, pyruvate, sialic acid, and other unknown substrates were found in the proteomic profile of Bin5. The different substrate preference between two dominant groups, flavobacteria and alphaproteobacteria, could reduce their competition in the coculture system.

10.1128/mBio.03261-19.9 TABLE S3 Exoproteome information for the five population bins of this study. Download Table S3, XLS file, 0.1 MB. Copyright © 2020 Zheng et al. 2020 Zheng et al. This content is distributed under the terms of the Creative Commons Attribution 4.0 International license.

A large complement of genes involved in the uptake and metabolism of inorganic nitrogen and phosphate compounds were also observed in the *Oricola* sp. Bin5 genome. In addition, three proteins (GlnAE and GltB) involved in ammonia assimilation were identified in the proteomic profile of *Oricola* sp. Bin5. Further, a urea ABC transporter (UrtA) protein and two proteins involved in urea decomposition (UreAC) were present in its proteome. The *Oricola* sp. Bin5 proteomic profile also contained two high-affinity phosphate ABC transporter proteins (PstSB) and an associated regulatory protein (PhoU). Last, two ABC transporter proteins involved in binding ferric iron were observed in the Bin5 proteome. Thus, the import of extracellular organic and inorganic nutrients was critical for *Oricola* sp. Bin5 growth. That also indicates *Oricola* sp. Bin5 may compete inorganic nutrients with *Synechococcus*, especially phosphate, in the coculture system.

### Exoproteome of *Synechococcus* sp. YX04-3 coculture.

A total of 150 proteins were identified (Tables S1B and S3) from the *Synechococcus* sp. YX04-3 coculture exoproteome which exhibited similar metabolic characteristics with cellular metaproteome (Text S1).

### Utilization of HMW complex biopolymers by *Flavobacteria*.

**FIG 4:**
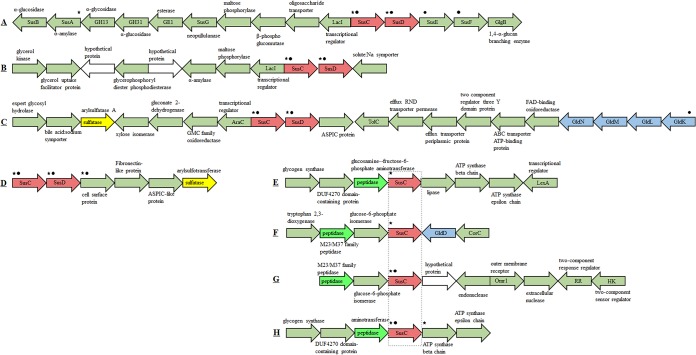
Multigene clusters encoding SusCD protein complexes (A, B, C, and D) and SusC-like proteins (E, F, G, and H) in the proteomic profiles of Bin3 and Bin2. A small solid black circle indicates that the proteins were detected in the cellular proteome, and a black star indicates that proteins were found in exoproteomes. (A and B) PULs targeting α-1,4-glucans; (C) PUL involved in the degradation of xylose-containing sulfated polysaccharides; (D) the PUL containing potential capacity for the degradation of sulfated polysaccharides/biopolymers; (E to H) gene clusters involved in the degradation of oligo- and polypeptides or proteins.

TBDT systems are outer membrane proteins involved in the uptake and utilization of HMW DOC molecules that cannot diffuse across membranes via porins. TBDTs targeting biopolymers (e.g., polysaccharides, proteins, and proteoglycan) are usually coupled to macromolecule substrate degradative enzymes (e.g., GHs and peptidases) to facilitate the efficient use of complex compounds (58, 59). SusC is a member of the TonB receptor family and is primarily responsible for importing oligosaccharides and polymers from the outer membrane into the periplasm, while SusD is an extracellular lipoprotein that is primarily involved in binding polysaccharides or other polymers at the outer cell membrane (60, 61). In addition to SusCD protein complexes, other SusC proteins were identified in the two flavobacterial proteomic profiles, suggesting that individual SusC proteins may independently mediate the transport of specific polymer types. Their upstream and downstream genes were next to genes involved in polymer degradation (e.g., GHs, amylases, peptidases, and sulfatases), motility, and attachment (Fig. 4).

The identified SusCD protein complex in the Bin3 proteome was encoded by genes associated with a polysaccharide utilization locus (PUL) (here defined as PUL01) that was predicted to target α-1,4-glucans like starch and glycogen (Fig. 4A and Fig. S4). The TonB-dependent receptor SusC operates in concert with the glucan-binding lipoproteins SusDEFG. SusDEF are active in the glucan-binding process, while SusG (GH13) catalyzes the initial degradation, SusC imports oligosaccharides into the periplasm, and SusA (GH13, also known as neopullulanse) and SusB (GH97, an α-glucosidase) further degrade oligosaccharides to glucose (62) (Fig. S4). The SusCD protein complex A in the Bin2 proteome, their encoding genes were adjacent to those coding for a maltose phosphorylase (GH65) and an alpha-amylase (GH13) (PUL02) (Fig. 4B), and therefore was also predicted to target α-1,4-glucans (61). The identified SusCD protein complex B encoding gene cluster in the Bin2 genome was located with genes upstream that encode a xylose isomerase and an arylsulfatase, suggesting the involvement of the former in the degradation of xylose-containing sulfated polysaccharides. In addition, genes located downstream encoded outer membrane efflux proteins and gliding motility proteins (GldKLMN) (Fig. 4C). Further, the SusCD protein complex C encoded in the Bin2 genome was associated with genes encoding cell surface proteins and a sulfatase, indicating the potential capacity for the degradation of sulfated polysaccharides/biopolymers (Fig. 4D). All of the identified individual SusC protein-coding genes in the Bin2 genome, except for one located at the end of the contig, were always adjacent to peptidases, suggesting that they specialized in the degradation of oligo- and polypeptides or proteins (Fig. 4E to H).

In addition, a xylan utilization gene cluster was detected in the *Muricauda* sp. Bin2 genome (Fig. S6A). An arabinogalactan utilization gene cluster was also present in the *Balneola* sp. Bin6 genome that comprised genes encoding an arabinogalactanase (*ganB*), β-galactosidase, galactokinase, and a SusCD system (Fig. S6B) (63, 64). However, these two PULs were not detected in the proteomic profiles of Bin2 and Bin3. Thus, these overall results suggest that the three flavobacterial populations in the coculture system displayed different polysaccharide degradation abilities.

10.1128/mBio.03261-19.6 FIG S6 Operon structure and gene composition of two-polysaccharide utilization loci (PUL) identified in the *Flavobacteria* Bin2 (A) and Bin6 (B) genomes, respectively. Download FIG S6, PDF file, 0.3 MB. Copyright © 2020 Zheng et al. 2020 Zheng et al. This content is distributed under the terms of the Creative Commons Attribution 4.0 International license.

The concentrations and organic molecular components of *Synechococcus* exudates vary over different growth phases or with physiological status (25, 26, 65). This variation in organic compound characteristics in turn influences the metabolic activities and population dynamics of heterotrophic bacteria in coculture systems. The two dominant flavobacterial populations, Bin2 and Bin3, should benefit from efficient strategies to metabolize specific HMW DOC compounds like glycogen, amylose, and maltodextrin that are released by *Synechococcus* in the exponential phase, relative to the flavobacterial Bin6 population. This is especially evident for the *Winogradskyella* sp. Bin3 population that could degrade α-1,4-glucans like glycogen that is biosynthesized by *Synechococcus* sp. YX04-3. This particular metabolic ability of the Bin3 population could partly explain its close association with *Synechococcus* cells.

### Oxidative stress and SODs.

Superoxide is an important reactive oxygen species (ROS) encountered by cells that can cause cellular damage and is especially problematic for oxygenic photoautotrophs. Previous studies have suggested that the removal of ROS is a key interaction between *Cyanobacteria* and their associated heterotrophic bacterial cells (13, 14, 66). Superoxide dismutases (SODs) and catalase or catalase-peroxidase enzymes play important roles in antioxidant defenses via the catalysis of superoxide (O^2−^) radicals to either O_2_ and H_2_O. As observed for *Prochlorococcus* strains or its close relative *Synechococcus* sp. WH8102, strain *Synechococcus* sp. YX04-3 lacked catalase or catalase-peroxidase genes, indicating it needs “helper” bacteria to reduce the ROS (46, 67, 68).

The catalase-peroxidase protein KatG catalyzes the decomposition of H_2_O_2_ to H_2_O and O_2_ and was detected in the cellular proteome of both the Bin2 and Bin5 populations. Two additional ROS stress proteins, Fe-SOD and alkyl hydroperoxide reductase subunit C protein (AhpC), were also present in the cellular proteome of Bin5. Further, a thiol-specific antioxidant family protein associated with thiol-[disulfide interchange was identified in the proteome of Bin4. Last, two of the bacterial populations, *Winogradskyella* sp. Bin3 and *Phycisphaera* sp. Bin4, that displayed attached or aggregate lifestyle preferences, contained SOD proteins in their exoproteomes. Despite limited data, these overall observations indicate that the removal of ROS is an active and important process in the coculture system.

### Vitamin B_12_ biosynthesis.

Many microbial species are auxotrophic for certain vitamins, lack specific vitamin biosynthetic pathways, and must therefore take up essential vitamins from their environments (69, 70). Vitamin B_12_ is an important cofactor for the activity of methionine synthase and is a core enzyme of cellular one-carbon metabolism, the production of the universal methyl donor *S*-adenosylmethionine, and for folate cycling required for DNA synthesis (71, 72).

Vitamin B_12_ is one of the most complex metabolites in natural environments and requires at least 19 separate enzymatic steps for its biosynthesis (69, 70). All of the heterotrophic bacterial genomic bins recovered, except the *Oricola* sp. Bin5 population, did not contain the key B_12_-independent methionine synthase (MetE) gene involved in biosynthesis, indicating that they all required exogenous vitamin B_12_. In contrast, the genomes of *Synechococcus* and *Oricola* sp. Bin5 contained all of the core genes for *de novo* cobalamin (vitamin B_12_) biosynthesis. Moreover, proteins involved in vitamin B_12_ biosynthesis (e.g., CobA, CobS, and CbiK) were observed in the proteomic profiles of *Synechococcus* sp. YX04-3 and *Oricola* sp. Bin5. Although the genes for vitamin B_12_ synthesis were not observed in the other genomes, genes involved in vitamin B_12_ transport were present in their genomes, including outer membrane receptors, binding proteins, permeases, and ATPase components. Two attached preferred bacterial populations, *Winogradskyella* sp. Bin3 and *Phycisphaera* sp. Bin4, lacked the core genes for thiamine (B_1_) and biotin (B_7_) biosynthesis, respectively. In addition, *Oricola* sp. Bin5 contained a set of ABC transport systems for importing thiamine instead of *de novo* biosynthesis in its genome. Thus, vitamin trafficking may contribute to interactions between populations in the coculture system and therefore partly account for the connectedness of microbial community networks in ocean environments (34, 73). These observations highlight the complex nature of interactions in these systems, as opposed to simple two-member interaction models.

### Nutrient cycling in the coculture system.

**FIG 5:**
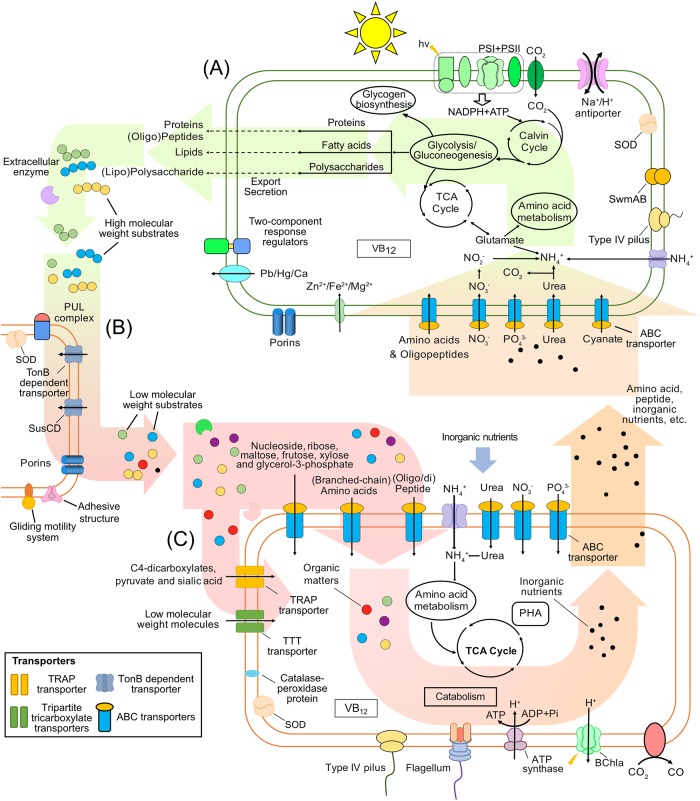
Schematic of inorganic and organic nutrient cycling in the *Synechococcus* (A), *Flavobacteria* (B) and *Alphaproteobacteria* (C) coculture system. The dominant detected metabolic processes observed for bacterial populations in the system are shown. *Synechococcus* is the primary driver of the coculture system functioning, and its dominant metabolic processes comprise inorganic nutrient uptake, photosynthesis, and organic matter biosynthesis and release. Two flavobacterial populations within the system corresponding to *Muricauda* and *Winogradskyella* strains, in addition to a member of the SM1A02 group exhibited specific propensities toward the initial degradation of complex compounds and biopolymers, as indicated by abundant TBDT, GH, and peptidase proteins in their proteomes. The alphaproteobacterium *Oricola* sp. Bin5 population mainly utilizes low-molecular-weight dissolved organic matter, including by-products from flavobacterial population metabolism, via their use of ABC, TRAP, and TTT transport systems. Thus, the bacterial members of the coculture system exhibit complementary metabolisms that operate in the degradation of *Synechococcus-*derived organic matter and nutrient cycling within the coculture system. PSI, photosystem I; TCA cycle, tricarboxylic cycle; VB_12_, vitamin B_12_; SOD, superoxide dismutase. The background arrows indicate the potential material and energy flows detected in the proteomic data.

The majority of organic matter in the coculture system was derived from photosynthetically fixed carbon by *Synechococcus*. Approximately 10% of the fixed carbon is released or leaked into the surroundings in the form of photosynthate (74–76) that then supports heterotrophic bacterial growth. The accumulation of photosynthate should thus become toxic to *Synechococcus* in nutrient-rich media and render inorganic nutrients unavailable within organic matter complexes (8). However, heterotrophs regenerate inorganic nutrients like NH_4_^+^, PO_4_^3−^, and Fe^2+/3+^ via their metabolism of organic matter, that *Synechococcus* can use (Fig. 5). The close associations between *Synechococcus* and heterotrophic bacteria suggest that these synergistic interactions are important drivers of interactions in coculture ecosystems.

The uptake of inorganic nutrients (e.g., NO_3_^−^, PO_4_^3−^, and Fe^2+^) is critical for photoautotrophic *Synechococcus* growth. Accordingly, transporter proteins for the above nutrients were abundant in both the cellular proteins and exoproteomes of *Synechococcus* (Fig. 5 and Fig. S3). In addition, approximately 10% of *Synechococcus* cellular proteins were associated with photosynthesis and CO_2_ fixation. Surplus photosynthetically fixed carbon could also be used to synthesize glycogen, a branched polysaccharide comprising glucose monomers that is an important mechanism for intracellularly storing carbon and energy reserves (49, 77). The multibranched glycogen biopolymer has a similar structure as amylopectin (a starch component) and is linked by α-1,4-glycosidic bonds on the stem chain and α-1,6-glycosidic bonds on branch chains. Many previous studies have shown that *Synechococcus* accumulates glycogen, although several *Synechococcus* strains harbor distinct α-polyglucans that have been termed semiamylopectin (78, 79). Proteins involved in the export of HMW biopolymers (e.g., polysaccharides, oligo/dipeptides, and lipids) were also identified in both the cellular and exoproteomic profiles of *Synechococcus*. Importantly, these compounds are also critical for the growth of coassociated heterotrophic bacterial populations.

The diverse nutrient transporters identified in the coculture system proteomes have different affinities and can be differentially regulated depending on nutrient concentrations and organic molecule complexity. Two of the flavobacterial strains appeared to be specialized in the degradation of complex HMW DOC molecules that exceed the typical 600- to 800-Da substrate range of normal porins (7, 80, 81) via the use of TBDT systems for direct attachment (Fig. S4). The alphaproteobacterial strain, *Oricola* sp. Bin5, appeared to be specialized in the uptake of labile LMW DOC including by-products of *Synechococcus* and flavobacterial growth via their expression of ABC and TRAP transporters and tripartite tricarboxylate transporters (TTTs) that were involved in monomer transport (Fig. S5). Thus, overall, it appeared that the flavobacterial and alphaproteobacterial strains acted synergistically to metabolize the organic matter released by *Synechococcus* (Fig. 5).

The proteomic profile of *Oricola* sp. Bin5 indicated that a large amount of nitrogen-rich [e.g., amino acids, (oligo)peptides, and proteins] and phosphorus-rich (e.g., glycerol-3-phosphate) compounds were present in the coculture system. These substrates were likely originally released as complex HMW compounds from *Synechococcus* that were further degraded by *Flavobacteria*. Nitrogen or phosphorus containing dissolved organic matter (DOM) molecules, typically as labile substrates, are preferentially utilized by heterotrophic bacteria. Specifically, heterotrophic bacteria use labile DOM to synthesize their biomass and respire it during energy conservation. The regenerated inorganic N and P nutrients, and especially those with P, would thus be returned to the culture medium, allowing the maintenance of a stable, healthy, and long-lived coculture system. The phase of coculture growth with *Synechococcus* and heterotrophic bacterial abundance stability after the 70th day may represent a temporary steady state of environmental variation via the utilization and regeneration of PO_4_^3−^, mitigation of oxidative stresses, and removal of ROS.

### Conclusions.

A long-term, 91-day cultivation experiment of the open ocean ecotype *Synechococcus* and its associated dominant heterotrophic bacterial populations revealed dynamic variation in their abundances, lifestyles, and organic matter metabolisms over the growth period. The *Synechococcus* and heterotrophic bacterial strains primarily exhibited synergistic interactions via nutrient exchange, reduction of ROS stress, and vitamin trafficking. Specifically, *Flavobacteria* and *Alphaproteobacteria* strains synergistically metabolized HMW *Synechococcus* photosynthate to LMW DOM and helped recycle inorganic nutrients. Indeed, the activities of the dominant coculture populations all contributed to the maintenance of the *Synechococcus* and heterotroph system. Light availability, inorganic and organic nutrient availability, and viral lysis and predation, together shape the cycling of matter and energy between photoautotrophs and heterotrophs in the upper ocean. To better understand these complex relationships *in situ*, additional research is needed to identify top-down and bottom-up controls that impact photoautotroph-heterotroph interactions.

## MATERIALS AND METHODS

### *Synechococcus* culture and abundance measurement.

*Synechococcus* sp. strain YX04-3, and its associated heterotrophic bacterial species, were isolated from the South China Sea using PRO2 liquid medium (25). In the present study, the YX04-3 coculture was incubated in triplicate at 25°C with 10 μmol photons · m^−2^ · s^−1^ in SN medium (82). *Synechococcus* and heterotrophic bacterial cell numbers in the cocultures were measured by flow cytometry as described previously (25).

### 16S rRNA gene sequencing and metagenome sequencing.

DNA from the 36 size-fractionated samples of the *Synechococcus* strain YX04-3 coculture were subjected to PCR amplification of 16S rRNA genes using primers targeting the bacterial V3-V4 hypervariable regions (515F [5′-GTGCCAGCMGCCGCGGTAA-3′] and 907R [5′-CCGTCAATTCMTTT RAGTTT-3′]) (83, 84). Sequence libraries were constructed using the NEBNext Ultra DNA library prep kit for Illumina (New England Biolabs, USA). The detailed protocols are described in Zheng et al. (25). The 16S rRNA gene sequence data were deposited in the NCBI Sequence Read Archive under the BioProject accession no. PRJNA498017.

Shotgun metagenomic sequencing was also conducted on the Illumina MiSeq platform (Illumina, San Diego, CA, USA) using the MiSeq V2 kit chemistry reagents with a 2 × 250 paired-end cycle sequencing run. Raw sequencing reads were trimmed and filtered and then *de novo* assembled by SPAdes (85). Assembled contig (>4 kbp) sequence properties, including GC content, tetranucleotide frequencies, and sequencing depth coverage were used to bin assembled contigs into draft genomes with the MaxBin 2.0 software program (86). Estimated genome sizes and completeness of the reconstructed genomes were then assessed using CheckM (87). The raw metagenomics sequence reads were deposited in the NCBI database under the BioProject accession no. PRJNA498190. The six metagenome bins were deposited in the NCBI database under the BioProject accession no. PRJNA497203.

### Metaproteomic analysis by liquid chromatography-tandem mass spectrometry (LC-MS/MS).

Metaproteomic analysis of the *Synechococcus* sp. YX04-3 coculture was conducted to elucidate the molecular mechanisms underlying population-level interactions. The 22nd day of cocultivation was chosen for targeted proteomic analysis because the number of *Synechococcus* cells was equivalent to the number of heterotrophic bacterial cells at this time point. To generate the metaproteomes, triplicate 120-ml liquid cultures were centrifuged (3,000 × *g* for 15 min at 4°C). The supernatant was then filtered through 0.22-μm-pore-size filter unit (Sterivex-GV, Millipore) and subjected to exoproteome analysis. The detailed protocol was conducted by the method of Christie-Oleza et al. (8) and supplied in the supplemental material. The mass spectrometry proteomics data have been deposited to the ProteomeXchange Consortium via the PRIDE partner repository with the data set identifier PXD015067.

### Data availability.

Sequence data were deposited in the NCBI Sequence Read Archive under BioProject no. PRJNA498017, PRJNA498190, and PRJNA497203.
