# Supplementary figures and images for: Metagenomic and Metaproteomic Insights into Photoautotrophic and Heterotrophic Interactions in a Synechococcus Culture
Source: mBio. 2020 Feb 18;11(1):e03261-19. doi: 10.1128/mBio.03261-19 (PMC7029141; doi:10.1128/mBio.03261-19)

**Figure S1**

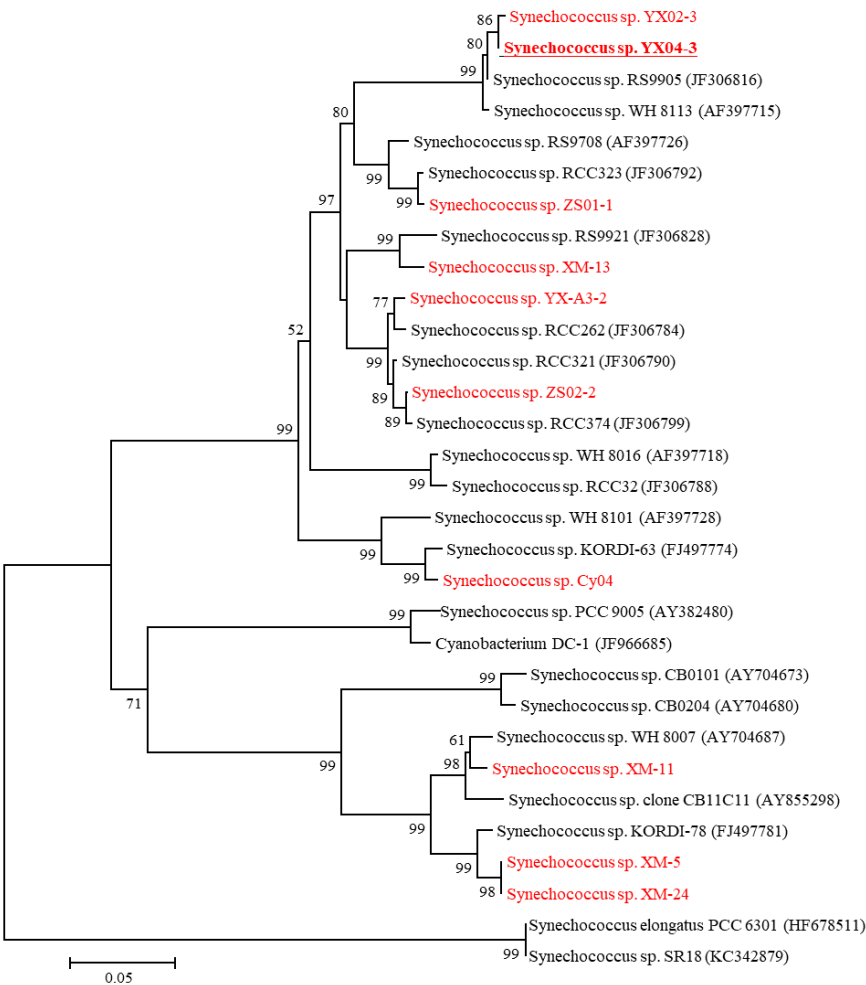

Supplement: FIG S1 [file mBio.03261-19-sf001.pdf]

Figure S2

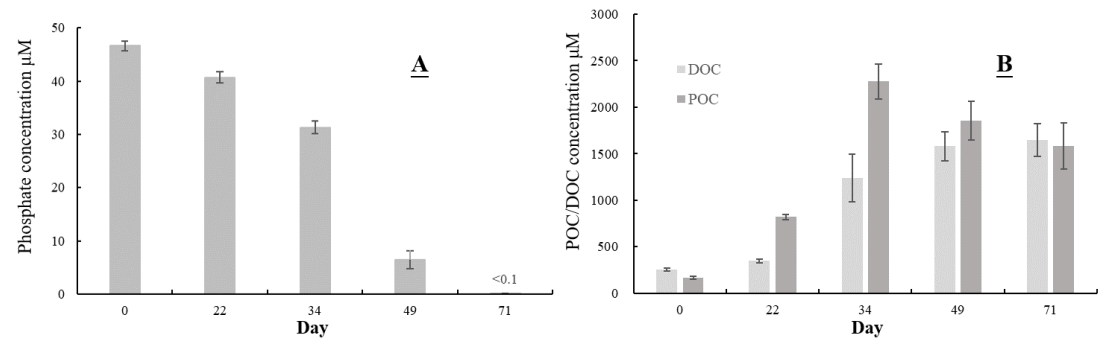

Supplement: FIG S2 [file mBio.03261-19-sf002.pdf]

**Figure S3**

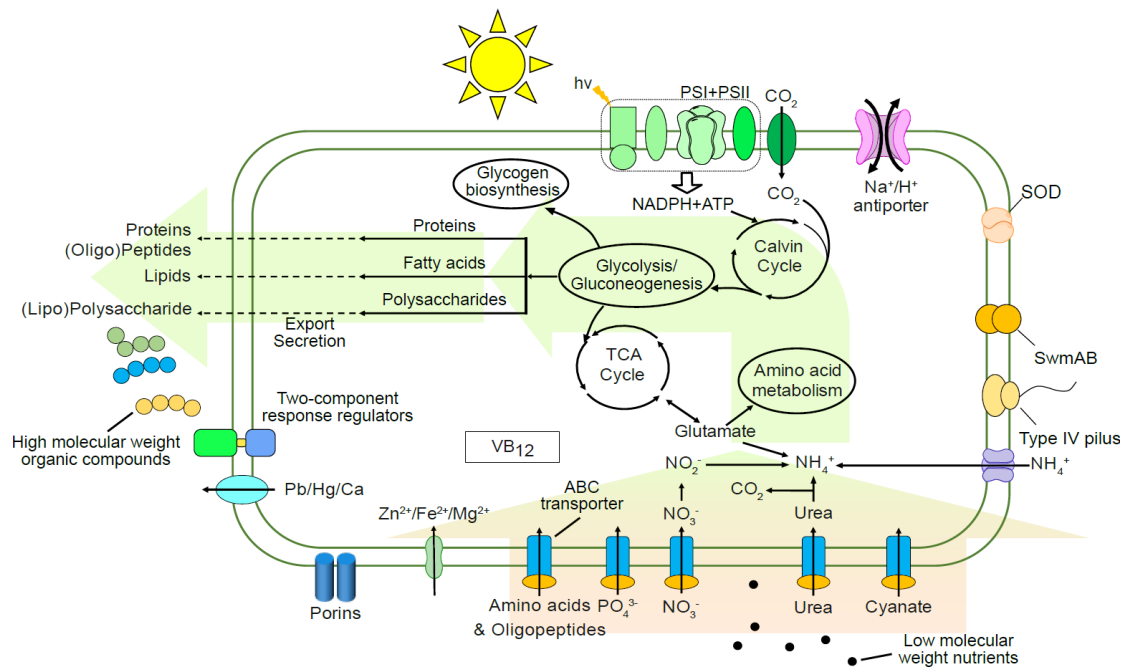

Supplement: FIG S3 [file mBio.03261-19-sf003.pdf]

Figure S4

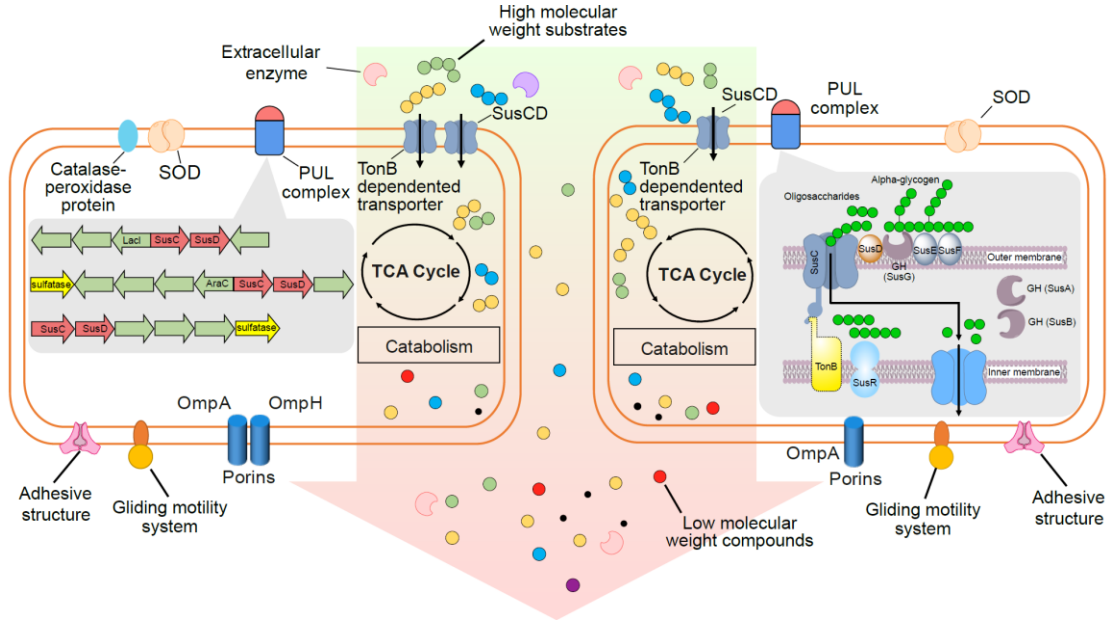

Supplement: FIG S4 [file mBio.03261-19-sf004.pdf]

**Figure S5**

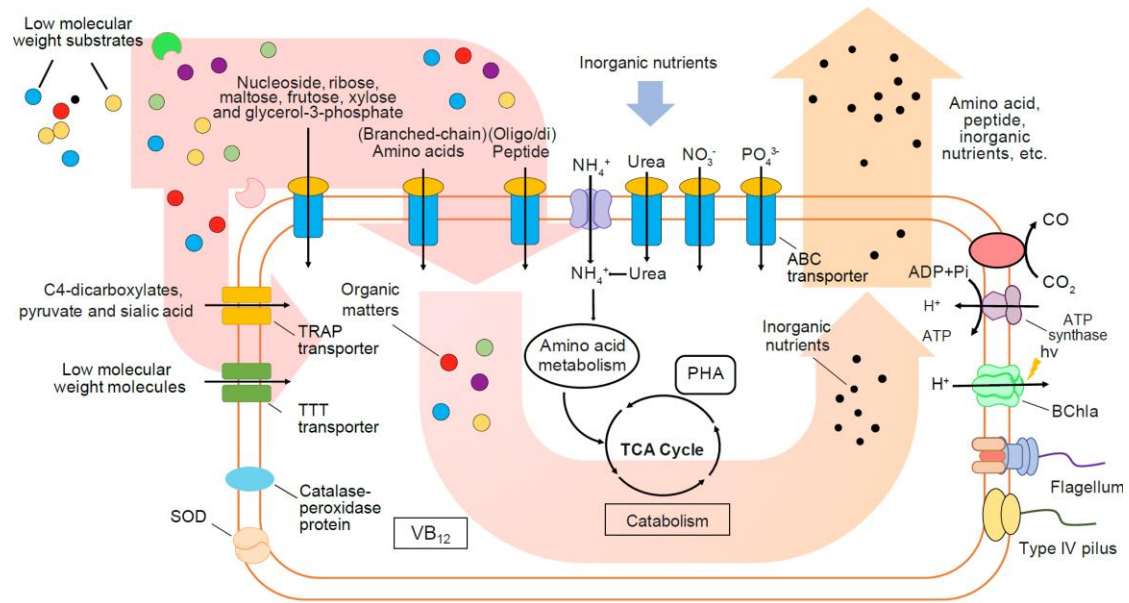

Supplement: FIG S5 [file mBio.03261-19-sf005.pdf]

Figure S6

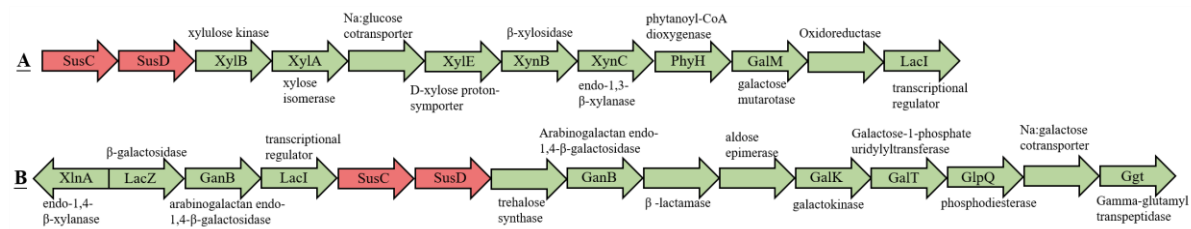

Supplement: FIG S6 [file mBio.03261-19-sf006.pdf]
