# Supplementary material for: Metagenomic and Metaproteomic Insights into Photoautotrophic and Heterotrophic Interactions in a Synechococcus Culture
Source: mBio. 2020 Feb 18;11(1):e03261-19. doi: 10.1128/mBio.03261-19 (PMC7029141; doi:10.1128/mBio.03261-19)
Supplement: TABLE S1 [file mBio.03261-19-st001.doc]

**Table S1A** The copy number of 16S rRNA gene in complete genomes

| Bacterial strains | GenBank Acc. No. | 16S copy no. |
| --- | --- | --- |
| *Muricauda lutaonensis* CC-HSB-11 | CP011071 | 2 |
| *Muricauda ruestringensis* DSM 13258 | CP002999 | 2 |
| *Winogradskyella* sp. RHA_55 | LT629774 | 3 |
| *Winogradskyella* sp. PC-19 | CP019332 | 2 |
| *Winogradskyella* sp. PG-2 | AP014583 | 2 |
| *Winogradskyella* sp. J14-2 | CP019388 | 2 |
| *Nitratireductor basaltis* RR3-28* | CP019044 | 1 |
| *Synechococcus* sp. WH 8020 | CP011941 | 1 |

*No member belonging to genus *Oricola* have been previously sequenced. Here we referred to genomic information for *Nitratireductor basaltis* RR3-28, which shares 96.9% (1282/1323) 16S rRNA gene sequence identity with *Oricola* sp. Bin5.

**Table S1B. Proteins identified in the proteomes of each co-culture population.**

| Population Bin | Total  ORFs* | Cellular proteome | | |  | Exoproteome | | |  | Relative abundance (16S rRNA) ** | |
| --- | --- | --- | --- | --- | --- | --- | --- | --- | --- | --- | --- |
|  |  | Identified proteins | Peptides | Matched spectra |  | Identified proteins | Peptides | Matched spectra |  | 0.22–3 μm | >3μm |
|  |  |  |  |  |  |  |  |  |  |  |  |
| *Synechococcus* sp. YX04-3 (Bin1) | 2,797 | 884 | 4,636 | 8,327 |  | 37 | 172 | 328 |  | 30.73% | 16.14% |
| *Muricauda* sp. Bin2 | 3,210 | 71 | 245 | 342 |  | 34 | 145 | 198 |  | 41.83% | 19.94% |
| *Winogradskyella* sp. Bin3 | 3,170 | 22 | 81 | 103 |  | 6 | 20 | 28 |  | 5.12% | 12.61% |
| *Phycisphaera* sp. Bin4 | 2,841 | 173 | 583 | 715 |  | 29 | 103 | 136 |  | 6.93% | 31.36% |
| *Oricola* sp. Bin5 | 3,639 | 896 | 3,971 | 5,905 |  | 44 | 202 | 308 |  | 8.03% | 1.82% |
| *Balneola* sp. Bin6 | 3,469 | 1 | 2 | 2 |  | - | - | - |  | 2.70% | 1.19% |

*ORFs represent the predicted open reading frames in each bin.

**Here means relative abundance in the total heterotrophic bacterial sequences at the 22^nd^ day corresponding to the metaproteome sampling time point.
