# Supplementary material for: Metagenomic and Metaproteomic Insights into Photoautotrophic and Heterotrophic Interactions in a Synechococcus Culture
Source: mBio. 2020 Feb 18;11(1):e03261-19. doi: 10.1128/mBio.03261-19 (PMC7029141; doi:10.1128/mBio.03261-19)
Supplement: TEXT S1 [file mBio.03261-19-s0001.doc]

**Supplementary Information for**

Meta-genomic and -proteomic insights into photoautotrophic and heterotrophic interactions in a *Synechococcus* culture

Qiang Zheng^1,2*^, Yu Wang^1,2^, Jiayao Lu^1,2^, Wenxin Lin^1,2^, Feng Chen^3^ and Nianzhi Jiao^1,2*^

Authors for correspondence:

Qiang Zheng: [zhengqiang@xmu.edu.cn](mailto:zhengqiang@xmu.edu.cn);

Nianzhi Jiao: [jiao@xmu.edu.cn](mailto:jiao@xmu.edu.cn)

**This file includes:**

- - - Genomic characteristics and lifestyle strategies of the dominant bacterial populations of the co-culture
    - The *Synechococcus* sp. YX04-3 co-culture system exoproteome
    - Detailed description about ‘Materials and Methods’
    - Supplementary References.

**Genomic characteristics and lifestyle strategies of the dominant bacterial populations of the co-culture**

***Synechococcus* sp. YX04-3 (Bin1).** The reconstructed genome of *Synechococcus* sp. YX04-3 was 2.46 Mbp in length (**Table 1**). A complete 16S rRNA gene was identified in the genome assembly that was nearly identical (1409/1411 nt) to that of the oligotrophic *Synechococcus* sp. strain WH8102, the genome of which was the first sequenced for marine unicellular *Synechococcus* (1). *Synechococcus* sp. YX04-3 preferred a free-living lifestyle (relative abundance of 30.2% in the 0.22–3 μm size fraction) rather than an attached/aggregate lifestyle (12.9% relative abundance in the >3 μm size fraction), which was consistent with observations from eutrophic *Synechococcus* co-culture systems (2) (**Figure 3**). *Synechococcus* relative abundances in the 0.22–3 μm size fraction decreased over the entire incubation period. In contrast, no obvious decreasing trends were observed in the >3 μm size fraction after the 4^th^ day of co-culture. However, it should be noted that the relative abundances of *Synechococcus* are heavily underestimated due to multiple 16S rRNA gene copies in the genomes of the dominant heterotrophic bacteria in the co-culture system (**Table S1A**). Consequently, we primarily focus on the distributional patterns among the different size fractions.

***Muricauda* sp. Bin2.** The genome corresponding to *Muricauda* sp. Bin2 exhibited a size of 3.10 Mbp and a GC content of 42.0% (**Table 1**). The *Muricauda* sp. bacterium genome encodes a proteorhodopsin system and a 16S rRNA gene with 94.6% (1323/1398 nt) sequence identity with the type strain *Muricauda pacifica* SW027^T^ (3). The level of 16S rRNA sequence divergence of that from *Muricauda* sp. Bin2 with other *Muricauda* cultivars (i.e., <95% nt identity) suggests that it represents a novel genus within the Flavobacteriaceae family. The *Muricauda* sp. was the most dominant heterotrophic bacterial population in the 0.22–3 μm size fraction, with higher relative abundance in that fraction compared to the >3 μm size fraction (**Figure 3**). Its relative abundances in the >3 μm size fraction remained relatively stable during the whole 91-day experimental incubation, with the exception of the 10^th^ day.

***Winogradskyella* sp. Bin3.** The *Winogradskyella* sp. Bin3 genome exhibited a size of 3.15 Mbp, and a GC content of 33.8%, which was the lowest among the six recovered genome bins (**Table 1**). The 16S rRNA gene (1429 nt length) from the *Winogradskyella* sp. Bin3 genome bin shared 97.2% nucleotide sequence identity with that of the type strain, *Winogradskyella wandonensis* WD-2-2^T^, which was isolated from a tidal flat (4). The genome of *Winogradskyella* sp. Bin3 also encoded a proteorhodopsin system. The *Winogradskyella* population was the most dominant bacterial taxa in the >3 μm size fraction and preferred an attached/aggregate lifestyle, as evinced by its distribution among the different size fractions. Its relative abundance in the >3 μm size fraction increased beginning at the 10^th^ day of cultivation until the end of the exponential phase, reaching a plateau during the stationary phase, and subsequently transitioning to a population decline period (**Figure 3**). Consequently, its relative abundances in the >3 μm size fraction coincided with the *Synechococcus* growth curve.

***Phycisphaera* sp. Bin4.** The *Phycisphaera* sp. Bin4 genome exhibited a size of 3.26 Mbp, with an average GC content of 61.2% (**Table 1**). A complete 16S rRNA gene sequence identified in the genome shared 81.9% (1152/1406 nt) nucleotide identity with that of the type strain *Phycisphaera mikurensis* NBRC 102666 that was isolated from the marine alga *Porphyra* sp. (5). Furthermore, the 16S rRNA gene sequence of Bin4 shared <97% nucleotide similarity with all other sequences in the GenBank database, with highest identity (90.4%) to the 16S rRNA gene sequence from a Planctomycete sp. GMD21C08 isolate that was obtained from the Sargasso Sea (6). Thus, the Bin4 population species represented a novel family within its class. The novel *Phycisphaera* sp. also exhibited a preference for an attached/aggregate lifestyle (**Figure 3**).

***Oricola* sp. Bin5.** The *Oricola* sp. Bin5 genome exhibited a size of 3.63 Mbp and a GC content of 64.8% (**Table 1**). A complete 16S rRNA gene was identified in the genome and its sequence shared 97.4% (1370/1407 nt) nucleotide identity with that of the type strain *Oricola cellulosilytica* CC-AMH-0 (7). The *Oricola* sp. Bin5 genome encoded the functional potential for a photoheterotrophic lifestyle, with a complete photosynthetic gene cluster (PGC) present. The *Oricola* sp. population exhibited a similar distributional pattern and lifestyle preference as *Synechococcus*, suggesting a close association between them. Its relative abundance in the 0.22–3 μm size fraction was much higher than in the >3 μm size fraction (**Figure 3**). Further, its relative abundance in the >3 μm size fraction did not exhibit obvious variation over the entire incubation period after the 4^th^ day. Its relative abundance in the exponential phase of the 0.22–3 μm size fraction also quickly decreased.

***Balneola* sp. Bin6.** The *Balneola* sp. Bin6 genome exhibited a size of 3.65 Mbp and a GC content of 40.0% (**Table 1**). In contrast to the other bacterial genomes, a proteorhodopsin system was not identified in its genome. The complete 16S rRNA gene within the Bin6 genome shared 94.4% (1334/1413 nt) nucleotide identity with that of the type strain *Balneola vulgaris* DSM 17893 that was isolated from the northwestern Mediterranean Sea (8). Although the *Balneola* sp. did not exhibit an obvious lifestyle preference across the different size fractions, it clearly responded to the *Synechococcus* decline phase (**Figure 3**). This response was much more evident in the 0.22–3 μm size fraction than in the >3 μm size fraction. These results indicate that the *Balneola* population preferred the free-living lifestyle after *Synechococcus* entered the decline phase.

**The *Synechococcus* sp. YX04-3 co-culture system exoproteome**

A total of 150 proteins were identified (**Table S1B, Table S3**) from the *Synechococcus* sp. YX04-3 co-culture exoproteome. Among these, 63 (42%) were involved in biological processes, 43 (29%) were associated with cellular components, and 66 (44%) were associated with molecular functions based on the GO annotations. The total numbers of protein originating from the populations corresponding to Bins 1, 2, 3, 4, and 5 ranged from 8 to 44 (**Table S1B)**, and are described in greater detail below.

**The *Synechococcus* sp. YX04-3 (Bin1) exoproteome.** A total of 37 proteins comprised the exoproteome profile of *Synechococcus* sp. YX04-3, of which hypothetical proteins with unknown functions comprised 21.7% (eight proteins). These results are consistent with previous studies suggesting that ‘hypothetical protein’ annotations within the exported protein fraction of *Synechococcus* proteomes were much higher than in their overall proteomes (9). Cell membrane proteins involved in inorganic (e.g., phosphorus, nitrogen, and iron) nutrient import and the export of organic compounds were also abundant in the exoproteomes of *Synechococcus*, including porins and ATP-dependent transporters (**Figure S3**). Specifically, four potential stress-induced phosphorus porins and two ABC periplasmic phosphate-binding transporter proteins were identified. In addition, two periplasmic proteins that bind urea and cyanate as part of ABC transport systems were present in the exoproteome of *Synechococcus*, as well as one glutamine synthetase protein that is involved in ammonia assimilation. Further, a TonB-like periplasmic binding protein responsible for ferric siderophore transport was also identified in the *Synechococcus* exoproteome, in addition to a cell surface protein required for swimming motility (SwmB). Lastly, three membrane proteins involved in exporting polysaccharides and other complex organic compounds were present in the *Synechococcus* exoproteome.

**The *Muricauda* sp. Bin2 exoproteome.** A total of 34 exoproteome proteins were associated with *Muricauda* sp. Bin2 that were primarily involved in the transport and metabolism of complex biopolymers, motility, and cellular attachment (**Figure S4**). Among these, three SusCD complexes and two more SusC-like proteins were identified in the Bin2 exoproteome that were all also in its cellular proteomic profile. In addition, two GH proteins and two S9 family peptidase proteins that are involved in the degradation and utilization of complex organic compounds were present in the Bin2 exoproteomic data. Further, the outer membrane OmpA and TPR proteins were also detected in its exoproteomic profile. Internalin proteins contain internal repeats or Ca^2+^-dependent adhesion motifs that participate in cellular attachment or aggregation processes (10). Three internalin proteins, with an average length of 3,825 amino acids, were observed in the Bin2 exoproteome data. In addition, two signal peptide proteins inferred to interact with environmental signals were detected, along with two proteins (GldJK) that are involved in gliding motility and a motor protein (MotB). The above proteins may allow the *Muricauda* cells to explore surfaces, including those of other cells. Consequently, these predicted secretory proteins involved in signal transduction, motility, cell adhesion (internalin, OmpA), and protein-protein interactions (TPR) could facilitate interactions between Flavobacteria and *Synechococcus* cells (11, 12).

**The *Winogradskyella* sp. Bin3 exoproteome.** The *Winogradskyella* sp. Bin3 population exhibited a clear preference for an attached or aggregate lifestyle over the co-cultivation period. The internalin protein described above that may function in cellular adhesion, was the most abundant protein in the proteomic profile of *Winogradskyella* sp. Bin3 (**Figure S4**). In addition, two types of super oxide dismutases (SODs) that use Cu/Zn and Mn as cofactors were detected in the Bin3 exoproteome. SODs are involved in protection against damage from reactive oxygen species (ROS). However, SOD proteins were also found in the cellular proteome of Bin2. A preference for the attached or aggregated lifestyle in association with *Synechococcus* could prompt an added pressure to reduce ROS damage for the Bin3 population, as compared to the Bin2 population. Three outer membrane proteins (SusCFG) that are involved in α-1,4-glucan (PUL01) transport and degradation were also observed in the Bin3 exoproteome.

**The *Phycisphaera* sp. Bin4 exoproteome.** A total of 29 proteins were identified in the *Phycisphaera* sp. Bin4 exoproteome, although most (21 proteins) were of unknown function. The relatively large amino acid sequence length (average ~800 aa length) of these proteins suggests that they may be involved in outer membrane attachment and aggregation, or otherwise the metabolism of complex compounds. For example, RHS repeat-associated proteins, RTX toxins, and related Ca^2+^-binding proteins were among the proteins of unknown function. Like the *Winogradskyella* Bin3 population, the *Phycisphaera* Bin4 population also preferred attached or aggregate lifestyles. In addition to the above, a Mn-SOD protein (Mn cofactor) was identified in the Bin3 exoproteome along with a thiol peroxidase associated with a thiol:disulfide interchange protein.

**The *Oricola* sp. Bin5 exoproteome.** A total of 44 exoproteome proteins belonged to the *Oricola* sp. Bin5 population, with 32 comprising substrate-binding membrane transporter proteins that play important roles in the uptake of available LMW organic matter and inorganic nutrients. Specifically, three types of transporter systems, ABC transporters, TRAP transporters, and tripartite tricarboxylate transporters (TTT) were identified in the exoproteomic profile of Bin5 (**Figure S5**). Of these, 25 ABC transporter and six TRAP-type substrate binding proteins were present that are involved in the transport of similar compounds as those detected in the cellular proteome of Bin5. In addition, a TTT-type substrate binding protein was uniquely present in the Bin5 exoproteome.

**Materials and Methods**

***Synechococcus* culture, cell harvesting, and DNA extractions**

*Synechococcus* sp. YX04-3 was isolated from seawater samples in the South China Sea around Yongxing island using PRO2 liquid medium (2), and a single *Synechococcus* ITS sequence was obtained after ~5-7-round dilutions. Seawater samples from the subsurface (1 m) were pre-filtered through 3.0-μm polycarbonate filters by gravity and the filtrates used for isolations. *Synechococcus* associated heterotrophs were self-selected natural assemblage. In the present study, the YX04-3 co-culture was incubated in triplicate at 25°C with 10 μmol photons·m^−2^·s^−1^ in SN medium (13). To collect size-fractionated samples, 6–8 mL of the *Synechococcus* liquid cultures were sequentially filtered through 3.0 and 0.22 μm pore-size polycarbonate filters (47 mm diameter; Millipore, USA) at a pressure of <0.03 MPa. To remove free-living cells from the 3 μm filters, a second, sterile 3 μm filter was placed on top of the first filter with cells, and the filters were turned over. Autoclaved 0.22 μm filtered artificial seawater was then used to wash the cells between the two filters and the two filters were then retained to represent the >3.0 μm fraction. Cell pellets and filters were flash frozen in liquid nitrogen and then stored at −80°C until DNA extraction. Cell morphologies were examined with transmission electron microscopy (TEM; JEM-1230, JEOL) using negative staining of cells with phosphotungstic acid The cells were collected during the stationary growth phase. DNA was extracted using a hot sodium dodecyl sulphate method with phenol/chloroform extraction, and precipitation with isoamyl alcohol, as previously described (14), followed by storage at −80^o^C until further use. The quality and quantity of extracted DNA was assessed using a Nano-Drop spectrophotometer (ND-2000; Thermo Fisher).

**Abundances of *Synechococcus* and heterotrophic bacterial cells**

*Synechococcus* and heterotrophic bacterial cell numbers in the co-cultures were measured using flow cytometry, as described previously (2). Briefly, an Epics Altra II flow cytometer (Beckman Coulter, Inc., Brea, CA, USA) equipped with an external quantitative sample injector (Harvard Apparatus PHD 2000; Instech Laboratories, Inc.) was used to quantify cells. *Synechococcus* cell numbers were determined from plots of side scatter versus red fluorescence and orange fluorescence versus red fluorescence (15). Total bacterial cells were enumerated, as described by Marie *et al.* (16) by staining cells with SYBR Green I and using plots of red fluorescence versus green fluorescence and side scatter versus green fluorescence. Subtraction of *Synechococcus* numbers from the total bacterial counts provided heterotrophic bacterial cell abundances. Flow cytometry data was analyzed using the EXPOTM 32 Multi-COMP software program (Beckman Coulter, Inc.).

**16S rRNA gene sequencing and sequence processing**

DNA from the 36 size-fractionated samples of the *Synechococcus* strain YX04-3 co-culture were subjected to PCR amplification of 16S rRNA genes using primers targeting the bacterial V3-V4 hypervariable regions (515F, 5’-GTGCCAGCMGCCGCGGTAA-3’ and 907R, 5’-CCGTCAATTCMTTT RAGTTT-3’) (17, 18). Sequence libraries were constructed using the NEBNext® Ultra™ DNA Library Prep Kit for Illumina (New England Biolabs, USA) according to the manufacturer's recommendations for NEBNext end preparation, adaptor ligation, size selection of adaptor-ligated DNA, PCR enrichment of adaptor-ligated DNA, and PCR amplification cleanups. Library quality was assessed using Qubit 2.0 Fluorometer (Thermo Scientific) and Agilent Bioanalyzer 2100 systems. The libraries were then sequenced on the Illumina MiSeq platform with paired-end 250 bp chemistry (Illumina, San Diego, CA, USA). Low quality reads (5-mer < Q20, length < 150 bp, or presence of ambiguous base-calls) were removed from the library, and the paired-end reads were combined using the FLASH software v1.2.7 program (19). Reads with homopolymer lengths >8 bp or >1 mismatch to the 5’ primer were removed from the libraries using QIIME v1.8.0 (20). Chimeras were then removed with USEARCH v5.2.236 (21) and UCLUST was used to cluster the remaining high quality reads into operational taxonomic units (OTUs) at a nucleotide identity cutoff of 97% (22). OTUs were then taxonomically classified against the RDP database (release 11.1; (23)) using the QIIME pipeline with default parameters (20). OTUs with a relative abundance of < 0.001% were removed to avoid overestimations of diversity, as described previously (24). The 16S rRNA gene sequence data was deposited in the NCBI Sequence Read Archive under the bioproject accession PRJNA498017.

**Metagenome sequencing and assembly**

Shotgun metagenomic sequencing was also conducted on the Illumina MiSeq platform (Illumina, San Diego, CA, USA) using the MiSeq V2 Kit chemistry reagents with a 2x250 paired-end cycle sequencing run. Metagenomic DNA library preparation was performed using the NEBNext® Ultra™ DNA Library Prep Kit (NEB, USA) following the manufacturer’s instructions. Raw sequence reads were trimmed and filtered using the criterion that all nucleotides within a read have a quality score equal to or above 20. Paired-end sequencing resulted in a total of 605.8 and 906.7 Mbp of sequence data from the exponential (22^nd^ day) and decline (71^st^ day) growth phase metagenomes, respectively. The raw reads were then *de novo* assembled with the SPAdes (25) assembler using the multiple k-mer option (command line parameters: -k 21,33,55,77,99,127–pe1-1–pe1-2 –careful). A total of 15.7 and 24.2 Mbp of assembled metagenome sequence (contigs > 1 Kbp) were produced for the *Synechococcus* cultures in the exponential and decline growth phases, respectively. The raw metagenomics sequence reads were deposited in the NCBI database under the BioProject accession PRJNA498190.

**Metagenomic binning, genome quality assessment, and gene annotation**

Contig (> 4kbp) sequence properties including GC content, tetranucleotide frequencies and sequencing depth coverage were used to bin assembled contigs into draft genomes with the MaxBin 2.0 software program (26). Estimated genome sizes and completeness of the reconstructed genomes were then assessed using CheckM (27). Gene prediction and annotation of the reconstructed genomes were conducted using the Rapid Annotation using Subsystem Technology pipeline (RAST; (28)). Functional annotations were also confirmed with BLASTp searches against the GenBank non-redundant (nr) protein database. Peptidase predictions were obtained by comparison against the MEROPS peptidase database (29), while GH and glycosyltransferase predictions were obtained by comparisons against the Carbohydrate Active Enzyme (CAZy) database (30). The six metagenome bins were deposited in the NCBI database under the BioProject accession PRJNA497203.

**Metaproteomic analysis by LC-MS/MS**

Metaproteomic analysis of the *Synechococcus* sp. YX04-3 co-culture was conducted to elucidate the molecular mechanisms underlying population-level interactions. The 22^nd^ day of co-cultivation was chosen for targeted proteomic analysis because the number of *Synechococcus* cells was equivalent to the number of heterotrophic bacterial cells at this time point. To generate the metaproteomes, triplicate 120 mL liquid cultures were centrifuged (3,000 x g for 15 min at 4°C). The supernatant was then filtered through 0.22 μm pore size filter unit (Sterivex-GV, Millipore) and subjected to exoproteome analysis. The centrifugation pellet was then collected to analyze the entire cellular proteomes. Pellets were washed with 0.2 M potassium phosphate buffer (pH 7.4), cells were added to STD buffer (4% SDS, 100 mM DTT, 150 mM Tris-HCl pH 8.0) and then incubated in a 99°C water bath for 5 min. To improve cell lysis, samples were sonicated for 10 s in 15 s intervals over 3 min. After centrifugation at 16,000 x g and 4°C over 30 min, the supernatants were transferred to new tubes for subsequent analysis. Proteins in the remaining mixtures were concentrated and purified via precipitation with trichloroacetic acid (31). Protein concentrations were determined using a BCA kit according to the manufacturer’s instructions.

Protein digestion was performed using a standard two-step in-solution digestion protocol. Briefly, the protein solution was reduced with 5 mM dithiothreitol for 30 min at 56°C, alkylated with 11 mM iodoacetamide for 15 min, then diluted by adding 100 mM Triethylammonium bicarbonate to a urea concentration below 2 M. Finally, trypsin was added to the solution at a trypsin-to-protein mass ratio of 1:50 for a first round of overnight digestion, and then again at a 1:100 ratio for a second round of digestion over 4 h. After trypsin digestion, tryptic peptides were dissolved in 0.1% formic acid (solvent A), and then directly loaded onto a home-made reversed-phase analytical column (15 cm length, 75 μm i.d.). The column was resolved using a gradient comprising solvents A and B (0.1% formic acid in 98% acetonitrile), with an increase from 6% to 23% of solvent B over 26 min, 23% to 35% over 8 min and then increasing to 80% over 3 min, followed by maintaining at 80% for the last 3 min. All of the above were conducted at a constant flow rate of 400 nL/min on an EASY-nLC 1000 UPLC system.

The recovered peptides were then analyzed on an NSI source linked to a tandem mass spectrometer (MS/MS) in a Q Exactive^TM^ Plus (Thermo) Mass spectrometer coupled online to the UPLC using a 2.0 kV electrospray voltage. The intact peptides were analyzed in the Orbitrap at a resolution of 70,000 with a m/z scan ranging from 350 to 1,800. Peptides were then chosen for MS/MS analysis using an NCE setting of 28, followed by detection of the fragments with the Orbitrap at a resolution of 17,500. The recovered MS/MS data were then processed using the Maxquant search engine software program (v.1.5.2.8) (32). The tandem mass spectra were searched against a database comprising all of the predicted ORFs from the two metagenomes that were concatenated with a reverse decoy database. Trypsin/P was used as the cleavage enzyme and up to two missing cleavages were allowed for the data set. A total of 102,911 spectra were obtained, with 1,478 spectra that were matched to our exoproteome database, in addition to 18,071 of 218,299 spectra resolved for the cellular proteome.

**Reference**

1. Palenik B, Brahamsha B, Larimer FW, Land M, Hauser L, Chain P, Lamerdin J, Regala W, Allen E, McCarren J. 2003. The genome of a motile marine *Synechococcus*. Nature 424:1037-42.

2. Zheng Q, Wang Y, Xie R, Lang AS, Liu YT, Lu JY, Zhang XD, Sun J, Suttle CA, Jiao NZ. 2018. Dynamics of heterotrophic bacterial assemblages within *Synechococcus* cultures. Appl Environ Microb 84:e01517.

3. O'Sullivan LA, Rinna J, Humphreys G, Weightman AJ, Fry JC. 2005. *Fluviicola taffensis* gen. nov., sp nov., a novel freshwater bacterium of the family *Cryomorphaceae* in the phylum 'Bacteroidetes'. Int J Syst Evol Micr 55:2189-94.

4. Lau SC, Tsoi MM, Li X, Plakhotnikova I, Dobretsov S, Wu M, Wong PK, Pawlik JR, Qian PY. 2006. Description of *Fabibacter halotolerans* gen. nov., sp. nov. and *Roseivirga spongicola* sp. nov., and reclassification of *Marinicola* *seohaensis* as *Roseivirga seohaensis* comb. nov. Int J Syst Evol Micr 56:1059-65.

5. Fukunaga Y, Kurahashi M, Sakiyama Y, Ohuchi M, Yokota A, Harayama S. 2009. *Phycisphaera mikurensis* gen. nov., sp nov., isolated from a marine alga, and proposal of *Phycisphaeraceae* fam. nov., *Phycisphaerales* ord. nov and *Phycisphaerae* classis nov in the phylum *Planctomycetes*. J Gen Appl Microbiol 55:267-75.

6. Zengler K, Toledo G, Rappe M, Elkins J, Mathur EJ, Short JM, Keller M. 2002. Cultivating the uncultured. P Natl Acad Sci USA 99:15681-6.

7. Hameed A, Shahina M, Lai WA, Lin SY, Young LS, Liu YC, Hsu YH, Young CC. 2015. *Oricola cellulosilytica* gen. nov., sp nov., a cellulose-degrading bacterium of the family Phyllobacteriaceae isolated from surface seashore water, and emended descriptions of *Mesorhizobium loti* and *Phyllobacterium myrsinacearum*. Anton Leeuw Int J G 107:759-71.

8. Urios L, Agogue H, Lesongeur F, Stackebrandt E, Lebaron P. 2006. *Balneola vulgaris* gen. nov., sp nov., a member of the phylum Bacteroidetes from the north-western Mediterranean Sea. Int J Syst Evol Micr 56:1883-7.

9. Christie‐Oleza JA, Armengaud J, Guerin P, Scanlan DJ. 2015. Functional distinctness in the exoproteomes of marine *Synechococcus*. Environ Microbiol 17:3781-94.

10. Schubert WD, Urbanke C, Ziehm T, Beier V, Machner MP, Domann E, Wehland J, Chakraborty T, Heinz DW. 2002. Structure of internalin, a major invasion protein of Listeria monocytogenes, in complex with its human receptor E-cadherin. Cell 111:825-36.

11. Gomez-Pereira PR, Schuler M, Fuchs BM, Bennke C, Teeling H, Waldmann J, Richter M, Barbe V, Bataille E, Glockner FO, Amann R. 2012. Genomic content of uncultured Bacteroidetes from contrasting oceanic provinces in the North Atlantic Ocean. Environ Microbiol 14:52-66.

12. Woyke T, Xie G, Copeland A, Gonzalez JM, Han C, Kiss H, Saw JH, Senin P, Yang C, Chatterji S. 2009. Assembling the marine metagenome, one cell at a time. Plos One 4:e5299.

13. Waterbury JB, Watson SW, Valois FW, Franks DG. 1986. Biological and ecological characterization of the marine unicellular cyanobacterium *Synechococcus*. Can Bull Fish Aquat Sci 71-120.

14. Fuhrman JA, Horrigan SG, Capone DG. 1988. Use of N-13 as tracer for bacterial and algal uptake of ammonium from seawater. Mar Ecol Prog Ser 45:271-8. https://doi.org/10.3354/meps045271.

15. Jiao NZ, Yang YH, Hong N, Ma Y, Harada S, Koshikawa H, Watanabe M. 2005. Dynamics of autotrophic picoplankton and heterotrophic bacteria in the East China Sea. Cont Shelf Res 25:1265-79.

16. Marie D, Brussaard CPD, Thyrhaug R, Bratbak G, Vaulot D. 1999. Enumeration of marine viruses in culture and natural samples by flow cytometry. Appl Environ Microb 65:45-52.

17. Lane D. 1991. 16S/23S rRNA sequencing. In ‘Nucleic acid techniques in bacterial systematics’.(Eds E Stackebrandt, M Goodfellow) John Wiley and Sons: Chichester, UK 115-75.

18. Stubner S. 2002. Enumeration of 16S rDNA of *Desulfotomaculum* lineage 1 in rice field soil by real-time PCR with SybrGreen (TM) detection. J Microbiol Meth 50:155-64.

19. Magoč T, Salzberg SL. 2011. FLASH: fast length adjustment of short reads to improve genome assemblies. Bioinformatics 27:2957-63.

20. Caporaso JG, Kuczynski J, Stombaugh J, Bittinger K, Bushman FD, Costello EK, Fierer N, Pena AG, Goodrich JK, Gordon JI. 2010. QIIME allows analysis of high-throughput community sequencing data. Nat Methods 7:335-6.

21. Edgar RC, Haas BJ, Clemente JC, Quince C, Knight R. 2011. UCHIME improves sensitivity and speed of chimera detection. Bioinformatics 27:2194-200.

22. Edgar RC. 2010. Search and clustering orders of magnitude faster than BLAST. Bioinformatics 26:2460-1.

23. Wang Q, Garrity GM, Tiedje JM, Cole JR. 2007. Naive Bayesian classifier for rapid assignment of rRNA sequences into the new bacterial taxonomy. Appl Environ Microbiol 73:5261-7.

24. Bokulich NA, Subramanian S, Faith JJ, Gevers D, Gordon JI, Knight R, Mills DA, Caporaso JG. 2013. Quality-filtering vastly improves diversity estimates from Illumina amplicon sequencing. Nat Methods 10:57-9.

25. Bankevich A, Nurk S, Antipov D, Gurevich AA, Dvorkin M, Kulikov AS, Lesin VM, Nikolenko SI, Pham S, Prjibelski AD, Pyshkin AV, Sirotkin AV, Vyahhi N, Tesler G, Alekseyev MA, Pevzner PA. 2012. SPAdes: A new genome assembly algorithm and its applications to single-cell sequencing. J Comput Biol 19:455-77.

26. Wu Y-W, Simmons BA, Singer SW. 2015. MaxBin 2.0: an automated binning algorithm to recover genomes from multiple metagenomic datasets. Bioinformatics 32:605-7.

27. Parks DH, Imelfort M, Skennerton CT, Hugenholtz P, Tyson GW. 2015. CheckM: assessing the quality of microbial genomes recovered from isolates, single cells, and metagenomes. Genome Research 25:1043-55.

28. Aziz RK, Bartels D, Best AA, DeJongh M, Disz T, Edwards RA, Formsma K, Gerdes S, Glass EM, Kubal M. 2008. The RAST server: Rapid annotations using subsystems technology. BMC Genomics 9:75-89.

29. Rawlings ND, Morton FR, Kok CY, Kong J, Barrett AJ. 2007. MEROPS: the peptidase database. Nucleic acids research 36:320-5.

30. Cantarel BL, Coutinho PM, Rancurel C, Bernard T, Lombard V, Henrissat B. 2008. The Carbohydrate-Active EnZymes database (CAZy): an expert resource for glycogenomics. Nucleic Acids Research 37:233-8.

31. Kaur A, Hernandez-Fernaud JR, Aguilo-Ferretjans MD, Wellington EM, Christie-Oleza JA. 2018. 100 Days of marine *Synechococcus-Ruegeria* pomeroyi interaction: A detailed analysis of the exoproteome. Environ Microbiol 20:785-99.

32. Cox J, Mann M. 2008. MaxQuant enables high peptide identification rates, individualized ppb-range mass accuracies and proteome-wide protein quantification. Nature biotechnology 26:1367-72.
